# Mms4 chromosomal association reveals functional relationships between meiotic crossover pathways in budding yeast

**DOI:** 10.1371/journal.pgen.1012097

**Published:** 2026-03-30

**Authors:** Amamah Farzlin Farnaz, Sameer Joshi, Praseetha Sarath, Girija Jogwar, Koodali T. Nishant

**Affiliations:** 1 School of Biology, Indian Institute of Science Education and Research Thiruvananthapuram, Trivandrum, India; 2 Center for High-Performance Computing, Indian Institute of Science Education and Research Thiruvananthapuram, Trivandrum, India; Dartmouth College Geisel School of Medicine, UNITED STATES OF AMERICA

## Abstract

Meiotic crossovers are generated from the repair of programmed DNA double-strand breaks (DSBs). In the budding yeast *Saccharomyces cerevisiae* and mammals, most crossovers are generated through the Class I pathway, involving the mismatch-repair related complex Msh4-Msh5, while a smaller fraction is produced by the Mms4-Mus81 endonuclease (Class II pathway). We present the first report on the genome-wide localization of the Mms4 protein during meiosis in *S. cerevisiae*. Surprisingly, Mms4 localization showed a trend towards weak DSB sites, unlike the localization of the Class I crossover protein -Msh5, which is biased towards strong DSB sites. This preference for weaker DSB hotspots was retained in a *msh5∆* mutant, arguing against competitive models of Mms4 and Msh5 association on meiotic chromosomes. The chromosomal association of Mms4 does not require the formation of meiotic DNA breaks but is facilitated by chromosome axis assembly. These results suggest Mms4 is primarily associated with chromosomal axis regions positioned near recombination intermediates. Mms4 binding is also largely insensitive to heterozygosity, unlike Msh5, consistent with its independence from recombination for localization. Together, these findings support a model in which Mms4-Mus81 enhances the robustness of meiotic recombination with a trend towards binding DSB hotspots that are weaker or are located in regions with sequence divergence that may be processed less efficiently by the Class I pathway.

## Author summary

Meiotic cells rely on carefully orchestrated DNA exchanges, called crossovers, to ensure genetic diversity and accurate chromosome segregation. In budding yeast and mammals, most crossovers are formed from the repair of DNA breaks by the Class I pathway, involving the mismatch repair-related proteins Msh4-Msh5. A second pathway (Class II), involving the Mms4-Mus81 complex, produces a smaller number of crossovers. In this study, we mapped for the first time the Mms4 binding sites across the entire budding yeast genome during meiosis. Surprisingly, Mms4 showed a trend towards binding weak DNA break sites, unlike Msh5 which favored strong DNA break sites. Strikingly, this trend was maintained even when Msh5 was absent. These studies suggest that while there is overlap in their distributions, the two pathways do not compete for the same DNA break sites. We also found that the chromosomal association of Mms4 does not require the formation of meiotic DNA breaks and is primarily associated with axis regions positioned near recombination intermediates. Mms4 binding is also relatively insensitive to sequence polymorphisms at DNA break sites, unlike Msh5. These findings indicate that Mms4-Mus81 provides resilience by repairing meiotic DNA breaks that are processed less efficiently by the Class I pathway.

## Introduction

Meiotic crossovers facilitate accurate segregation of homologous chromosomes at the first meiotic division (MI). Failure in this process may result in aneuploidy, which is responsible for congenital genetic diseases like Down syndrome in humans [1]. Crossovers along with sister chromatid cohesion establish physical connections between homolog pairs such that homologs are oriented towards opposite poles of the bipolar spindle, ensuring accurate segregation [2]. Meiotic crossovers arise from programmed DNA double-strand breaks (DSBs) initiated by Spo11 and accessory proteins during the prophase stage of Meiosis I [3–6]. These breaks are repaired preferentially using the homologous chromosomes as a template to generate either crossovers or non-crossovers [7–10]. Crossover formation is tightly regulated to ensure at least one crossover per homologous pair of chromosomes, known as an obligate crossover [11]. Further, crossover homeostasis ensures a consistent number of crossovers at the expense of non-crossovers when DSB frequencies are reduced [12].

In the budding yeast, during meiotic prophase, chromosomes are organized into loops anchored by axial elements composed of Red1, Hop1 and the meiotic specific kleisin cohesin subunit and Rec8 [13–20]. DSB-promoting proteins are associated with both loop and axis regions, supporting the idea that DSBs form in the context of this loop-axis organization [21,5]. In *S. cerevisiae*, the DSBs are resolved into crossovers using two main pathways. Approximately 70% of crossovers (Class I) form through a ZMM dependent pathway, comprising the mismatch repair-related Msh4-Msh5 complex, along with Zip1–4, Mer3, and Spo16 [22–31]. Additional pro-crosssover factors in the Class I pathway include the STR complex (Sgs1, Top3, Rmi1) and the Exo1-MutLγ (Mlh1-Mlh3) complex [9,10,32–39]. A smaller set of crossovers (Class II, approximately 30%) is formed via structure-specific endonucleases such as the Mms4-Mus81 complex, Yen1, and Slx1–Slx4 [32,35 40–43]. Class I crossovers are interfering, ensuring evenly spaced crossovers on each chromosome [44,45], whereas Class II crossovers are non-interferring [41]. Out of ~170 DSBs, around 90 are repaired into crossovers in *S. cerevisiae* [21]. The remaining DSBs are processed into non-crossovers or repaired by inter-sister recombination that do not yield either crossovers or non-crossovers [9,46–48].

The Mms4-Mus81 complex is homologous to the Rad1-Rad10 (XPF-ERCC1) endonuclease complex involved in nucleotide excision repair [49–51]. The Mms4-Mus81 complex cleaves branched DNA structures, such as 3’ overhangs, D loops and nicked and fully ligated Holliday junctions, *in vitro* [52,53]. It plays a major role in processing stalled replication forks and Holliday junctions in mitotic cells [49,51,54], but a minor role in processing meiotic joint molecules [55]. In *mms4* mutants, meiotic recombination intermediates accumulate, leading to reduced spore viability (~50%) and a modest decrease in crossover formation (20–30%) [32,40,41,55,56]. Furthermore, crossover defects are predominantly observed on small chromosomes [41]. These effects become more severe when Yen1 is absent, suggesting Yen1 acts as a backup to resolve joint molecules post meiosis I that escape Mms4-Mus81 [55]. Activation of Mms4-Mus81 is regulated through Cdc5-dependent phosphorylation of Mms4, enhancing its nuclease activity in late meiotic prophase to resolve any remaining joint molecules [55]. This regulatory mechanism ensures that Mus81-Mms4 acts as a late-stage resolvase in meiosis.

Although the localization of Class I crossover proteins on meiotic chromosomes has been extensively studied [57–61], the localization of Class II crossover factors (Mms4-Mus81) during meiosis remains unclear. Here, we provide the first genome-wide analysis of Mms4 binding during meiosis in *S. cerevisiae*. Our findings reveal features of Mms4 association on meiotic chromosomes that are distinct from those of the Class I crossover protein Msh5. These include a trend for binding weaker DSB hotspots, reduced peak width, association with meiotic chromosomes independent of Spo11 activity, lack of competition with Msh5 for chromosomal association, and reduced sensitivity to genomic heterozygosity. Further, the strong association of Mms4 with axis regions may suggest storage locations prior to activation that are strategically positioned near recombination intermediates throughout the genome [5,62,63]

## Materials and methods

### Yeast strains and media

All yeast strains used in this study are derived from *S. cerevisiae* backgrounds SK1, S288c or YJM789 and *Saccharomyces mikatae*. The strains and genotype are listed in S1 Table. Yeast cultures were grown at 30°C in either yeast extract–peptone–dextrose (YPD) or synthetic complete medium, following established procedures [64–66]. Mutant strains were created using standard transformation methods [67]. The growth media included geneticin (Invitrogen), nourseothricin (Werner BioAgents, Germany), or hygromycin (Sigma) at recommended concentrations [68] to select for transformed strains. Mms4 was C-terminally tagged with a 9xMyc cassette in wild type and mutant strain backgrounds using a Polymerase Chain Reaction (PCR)-based method described by Janke et al., [69]. All transformed strains were confirmed via PCR with flanking primers. Sanger sequencing was performed to verify the Mms4-9xMyc tagged strain.

### Tetrad analysis

The Mms4-9xMyc diploid strain was patched on a synthetic complete medium and incubated for 4 h according to the zero-growth mating protocol [32]. The resulting diploids were patched on sporulation medium and incubated for 48 h at 30°C to obtain tetrads. Tetrads were dissected on a synthetic complete medium using a Zeiss dissecting microscope.

### Meiotic synchronization

The yeast strains were synchronized as described in [70]. Strains were individually streaked on YPD medium. A single colony from each strain was inoculated into 5 mL of YPD liquid medium and incubated overnight at 30°C. The cultures were first grown in pre-sporulation medium (SPS: 0.5% yeast extract, 1% peptone, 0.67% yeast nitrogen base, 1% potassium acetate, and 0.05 M potassium biphthalate) before transferring into sporulation medium (SPM: 2% potassium acetate, 3.2 µl/ml amino acid mix, and 0.001% polypropylene glycol). 2 ml of meiotic cultures post-meiotic induction were collected for each strain at different time intervals in a chronological sequence (0, 2, 4, 6, 8, 10, 12, and 24 hours). Nuclei were stained with 4’,6-diamidino-2-phenylindole (DAPI) and visualised using an Olympus fluorescence microscope. The sporulation efficiency of the strains was checked by monitoring the nuclear divisions as they progressed through Meiosis I and Meiosis II. A hundred cells were analyzed from two replicates at each time point.

### Protein extraction and Western blotting

The trichloroacetic acid precipitation method was used to prepare the protein extracts from 2 ml of meiotic culture as described in Foiani et al., [71]. Sodium Dodecyl Sulfate–Polyacrylamide Gel Electrophoresis (SDS-PAGE) gel (8%) was used for electrophoresis, followed by blotting and probing of the target protein-Mms4. The following primary antibody dilutions were used: mouse anti-Myc antibody (Sigma, M4439, 1:1000); mouse anti-Pgk1 antibody (Novus Biologicals, NBP1–33685, 1:30,000). Anti-mouse HRP (Jackson ImmunoResearch, 715-035-150) was used as a secondary antibody at a dilution of 1:10,000 for Mms4-9xMyc and 1:40,000 for Pgk1.

### Calibrated chromatin immunoprecipitation

Synchronized meiotic cultures of *S. cerevisiae* strains- homozygous SK1 wild type, mutants- *red1∆*, *spo11∆*, *msh5∆*, the hybrid S288c-sp/YJM789 and *S. mikatae* strain were prepared as described [60]. Preparation of cell lysate, incubation with beads, and elution were performed as described earlier [60,61]. In brief, meiosis was induced in 200 ml liquid cultures at 30**°**C. 50 ml cultures were collected at selected time points (3–5 hrs) and then centrifuged and washed with Tris-buffered saline (TBS) buffer. Cell pellets were collected for both *S. cerevisiae* and *S. mikatae* strains and mixed in a 1:10 (*S.mikatae*: *S.cerevisiae*) ratio for performing calibrated Chromatin immunoprecipitation (ChIP) [61,72]. Cells were suspended in lysis buffer containing Aprotinin, Phenylmethylsulfonyl fluoride (PMSF) and Protease Inhibitor Cocktail (Merck, 11873580001), mechanically lysed using a mini bead beater and sonicated to yield DNA fragments of ~300–400 bp in length. The cell lysate was incubated overnight at 4**°**C with magnetic Protein G Dynabeads (Thermo Fisher, 10003D) bound by an Anti-Myc antibody. The Mms4-9xMyc, along with bound DNA was eluted from the magnetic beads using an elution buffer (50 mM Tris HCl at pH 8.0, 50 mM EDTA [Ethylenediaminetetraacetic acid] and 1% SDS) at 65**°**C. The DNA was released with Proteinase K digestion, eluted and used for ChIP-qPCR (quantitative polymerase chain reaction) and ChIP-sequencing. Untagged wild-type strains were used as a negative control. The immunoprecipitated DNA from two biological replicates for each time point was sequenced on the Illumina NovaSeq platform at Macrogen, South Korea (50 bp paired-end).

### Calibrated ChIP-seq analysis

The calibrated ChIP-seq data were analyzed as described by [72]. The quality of raw reads was checked using FastQC (https://www.bioinformatics.babraham.ac.uk/projects/fastqc/) software. The raw reads were aligned separately to *S. cerevisiae* S288c (version 64-1-1, 2011) and *S. mikatae* IFO1815 (GCF_947241705.1) genomes using Bowtie2 (version 2.3.5.1) [73]. Uniquely mapped *S. cerevisiae* reads were extracted by aligning unmapped *S. mikatae* reads to the *S. cerevisiae* genome. Similarly, uniquely mapped *S. mikatae* reads were extracted by aligning unmapped *S. cerevisiae* reads to the *S. mikatae* genome. For each replicate, uniquely mapped *S. cerevisiae* reads were divided by uniquely mapped *S. mikatae* reads to obtain scaling factor 1 (SF1). The SF1 was further divided by the untagged sample coverage to obtain scaling factor 2 (SF2). To calculate the calibrated Mms4 read depth, the S288c genome was partitioned into 10 bp bins and the uniquely mapped *S. cerevisiae* Mms4 reads in those bins were estimated. These were multiplied by SF2 to obtain the normalised Mms4 read depth. The normalized Mms4 reads from the untagged sample were then subtracted from the normalized Mms4 reads corresponding to the experimental (ChIP) samples to obtain calibrated Mms4 read depths across the genome. The calibrated Mms4 read depths were further averaged across two replicates for each sample, followed by genome-wide smoothing using the ksmooth function in R with a bandwidth of 1 kb. Plotting was performed in R. Spo11 oligo data were obtained from Pan et al., [21] and Thacker et al., [70], whereas Red1 data were obtained from Sun et al., [19]. Centromere locations were extracted from SGD (Saccharomyces Genome Database).

### Peak calling

The MACS2 (model-based analysis for ChIP-Seq, version 2.2.7.1,[74]) algorithm was used for genome-wide Mms4 peak calling. Peak calling was performed as described in Dash et al., [61]. Mms4 peaks were called from a pooled dataset of two replicates for each sample. Peaks with P value > 10^-5^ were filtered out to obtain genuine Mms4 peaks. Mms4 peaks were annotated with Spo11, Red1, and centromere locations as described in Dash et al., [61].

### ChIP-qPCR

ChIP-qPCR was performed on the immunoprecipitated DNA in both wild type and mutant backgrounds. Mms4 enrichment was calculated at specific genomic loci, including DSB hotspots (*BUD23*, *ECM3*, *CCT6*, *TEL01L*, *TEL05R*, and *FUN12*), chromosome axis-associated sites (*Axis I*, *Axis II*, and *Axis III*), centromeric regions (*CEN III* and *CEN VIII*), and a known DSB coldspot (*YCR093W*). *Axis I* (Chromosome XV: 781105–781228 bp) represents a discrete axis region devoid of nearby DSB hotspots or coldspots; *Axis II* (Chromosome IV: 847384–847560 bp) is located adjacent to the DSB hotspot *CCT6*; and *Axis III* (Chromosome III: 266308–266448 bp) lies near the DSB coldspot *YCR093W*. DNA enrichment at each site was normalized to input DNA and further normalized to the cold spot and the untagged control. Two independent biological replicates were analyzed for each genotype.

### Meiotic co-immunoprecipitation (Co-IP)

*S. cerevisiae* SK1 cells were induced into meiosis and harvested at the desired meiotic time points. Sample collection and extract preparation were carried out as described previously by Pannafino et al., [75]. For immunoprecipitation, extracts were incubated with 10 µL of anti-Myc antibody (Sigma, M4439) to pull down Mms4-9xMyc. Protein G Dynabeads (Thermo Fisher Scientific, 10003D) was used to capture antibody–protein complexes according to the manufacturer’s protocols. Immunoprecipitates were washed and ran on SDS–PAGE gel, followed by Western blotting. The anti-Myc antibody (Sigma, M4439) was used for both immunoprecipitation and subsequent Western blot analysis of Mms4-9xMyc. Red1 was detected using a native antibody generously provided by Prof. K. Muniyappa, Indian Institute of Science, Bangalore. Input fractions and immunoprecipitated fractions were loaded as indicated in the figure legends.

### Statistical tests

The Wilcoxon ranked-sum test was used for violin, box and density distribution plots. Karl Pearson’s correlation coefficient was used to calculate the correlation values for linear regression analysis.

## Results

### Mms4 binds to DSB hotspots, chromosome axes, and centromeres on meiotic chromosomes

**Fig 1:**
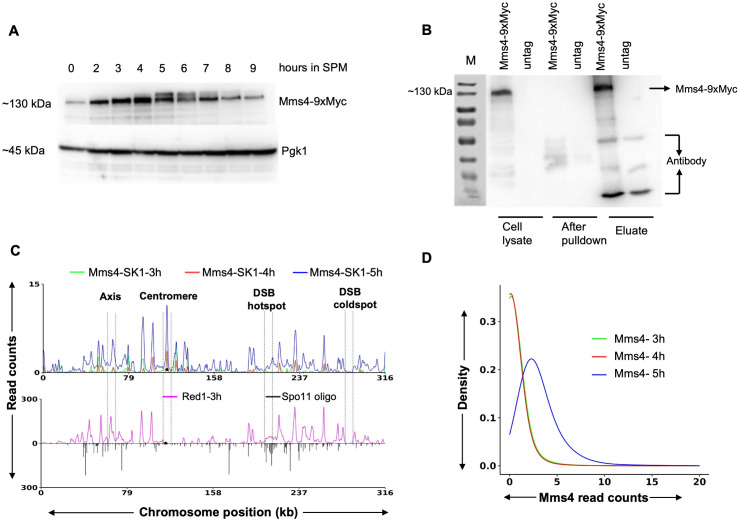
Expression and genome wide binding pattern of the Mms4-9xMyc protein. **A)** Western blot analysis of Mms4-9xMyc expression from 0 to 9 hours after meiotic induction. Pgk1 serves as a loading control. **B)** Mms4-9xMyc ChIP using the anti-Myc antibody in synchronized wild-type meiotic cultures. Lanes 1, 3, and 5 represent the Mms4-9xMyc strain; lanes 2, 4, and 6 represent the untagged wild-type strain. M: molecular weight marker. **C)** Calibrated ChIP-seq profile showing Mms4-9xMyc binding on chromosome III at 3,4 and 5 hours post meiotic induction. Red1 and Spo11 data are from [19] and [21], respectively. The black circle marks the centromere. Dotted lines indicate centromeric region, axis region, DSB hotspot (*BUD23*), and DSB coldspot (*YCR093W*). **D)** Density plot of Mms4 ChIP-Seq read counts at Mms4 peak locations at 3h, 4h, and 5h post meiotic induction. The X-axis represents Mms4 binding strength, measured by the number of Mms4 reads in 10 bp bins at each peak, while the Y-axis indicates the probability density for a given number of Mms4 reads in the genomic bins.

To determine Mms4 binding sites genome-wide, we tagged the Mms4 protein using a 9xMyc tag. The spore viability of Mms4-9xMyc was 96.5% compared to 99.6% for wild type (S1A Fig). The sporulation efficiency of the Mms4-9xMyc tagged strain was the same as that of the wild-type untagged strain, achieving 100% sporulation in 24 hours (S1B Fig). These results suggest that the introduction of the 9xMyc tag on Mms4 did not change the meiotic spore viability or sporulation kinetics. The expression profile of Mms4 during meiosis was monitored in synchronised meiotic cultures, with samples collected at various time points (0, 2, 3, 4, 5, 6, 7, 8 and 9 hours) post-induction of meiosis. The protein extracts were prepared using the trichloroacetic acid precipitation method followed by Western blotting using an anti-Myc antibody (Fig 1A). The housekeeping gene Pgk1 was used as a loading control. Peak Mms4 expression was observed at ~4–5 hours post-meiotic entry, with the appearance of the slower migrating activated phosphorylated form at 5 hours [55]. The molecular weight of Mms4 is ~ 78 kDa, and the expected size of the tagged version is around 90 kDa. 9xMyc-tagged Mms4 migrated at a position of ~130 kDa (Fig 1A), as has been previously observed [52].

**Fig 2:**
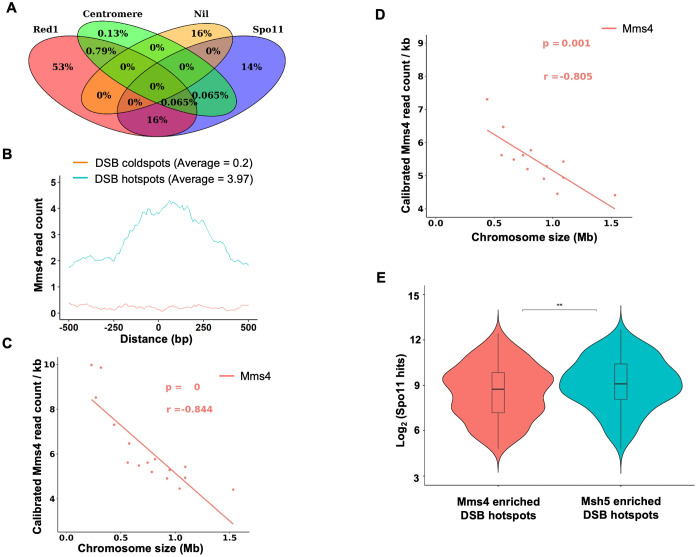
Analysis of Mms4 binding during meiosis. **A)** Venn diagram showing the distribution of Mms4 enrichment (5h) across DSB hotspots (Spo11), chromosome axes (Red1), and centromeres, based on at least 1 bp overlap with Mms4 peak coordinates (1528 Mms4 peaks, S2A Table). ‘Nil’ represents genomic regions that do not fall into any of these categories. **B)** Mms4 binding at top 25 DSB hotspots (based on Spo11 oligo counts from [21]) and 25 DSB coldspots [39] in SK1 wild-type strain at 5 hours post meiotic induction. The Y-axis represents the average Mms4 read count using a 10 bp window size. The X-axis spans ±0.5 kb from the centre of each hotspot or coldspot. **C, D)** Mms4 read density plotted as a function of chromosome size using a linear regression model (excluding the highly repetitive chromosome XII rDNA coordinates from 451418- 489465 bp [76]). Read density is expressed as reads per million per kilobase (RPM/kb). **C)** includes all chromosomes, while **D)** excludes short chromosomes (I, III, **VI)**. The *p*-values and *r*-values represent the statistical significance and the strength of the correlation, calculated using Karl Pearson’s correlation coefficient. **E)** Violin plot representing log_2_ of Spo11 reads [21] at DSB hotspots enriched specifically for Mms4 or Msh5. The horizontal line indicates the median value. ** indicates p < 0.01 (Wilcoxon rank-sum test).

As peak Mms4 expression was observed at approximately 4–5 hours, calibrated Mms4 ChIP was performed in the wild-type SK1 strain using an anti-Myc antibody at 3, 4, and 5 hours post-meiotic induction from two biological replicates to determine the Mms4 binding profile genome-wide. Calibration was performed using an *S. mikatae* spike in sample (Materials and Methods). The immunoblot analysis of Mms4 ChIP showed a band at the expected size of 130 kDa in the lysate and eluate fractions of the Mms4-9xMyc tagged strain. (Fig 1B). No band at the expected size for Mms4-9xMyc (130 kDa) was observed in the untagged lysate or eluate fractions (Fig 1B). The Mms4 ChIP-Seq data was normalized using the untagged strain. The representative plot on chromosome III shows Mms4 enrichment at chromosomal axis regions, centromeres and DSB hotspots (Fig 1C). Mms4 binding plots for other chromosomes are shown in S2A Fig. Genome wide analysis showed that maximum Mms4 binding was observed at 5h compared to 3h and 4h (Fig 1C and 1D). A total of 1528 Mms4 peaks were observed in the wild type at 5h (S2A Table). Quantitative analysis of the Mms4 peak locations shows the distribution of Mms4 across axes, DSB hotspots and centromeres (Fig 2A). Binding outsides of these regions was classified as Nil (Fig 2A). An analysis of Mms4 binding at the top 25 DSB hotspots [21] compared to 25 DSB coldspots [39] showed enhanced binding at DSB hotspots compared to the coldspot regions (Fig 2B**).**

Since DSB hotspot density shows a negative correlation with chromosome size [21,77], we investigated whether Mms4 read density exhibits a similar correlation. Linear regression analysis of Mms4 read density was performed both with and without the three smallest chromosomes (I, III, and VI), which are known to have unusually high Spo11 density [77]. When all chromosomes were included, the Mms4 read density showed a negative correlation with chromosome size (r = -0.84, p = 0), (Fig 2C). Even after excluding the three smallest chromosomes, the negative correlation with chromosome size was maintained (r = -0.80, p = 0.001, Fig 2D). These results suggest that Mms4 binding is negatively correlated with chromosome size consistent with short chromosomes having more Mms4 dependent crossovers [41]. This is also similar to the Msh5 binding pattern, where negative correlation was maintained even after excluding the smallest three chromosomes [60]. We tested whether, Mms4 binding shows regional variation along the chromosome. DSB hotspots near the centromeres were Mms4 enriched (and Msh5 depleted) on 11 of the 16 chromosomes (S3A Fig). This pattern was however, not statistically significant (S3B Fig).

### Mms4 shows a trend towards binding weak DSB hotspots

**Fig 3:**
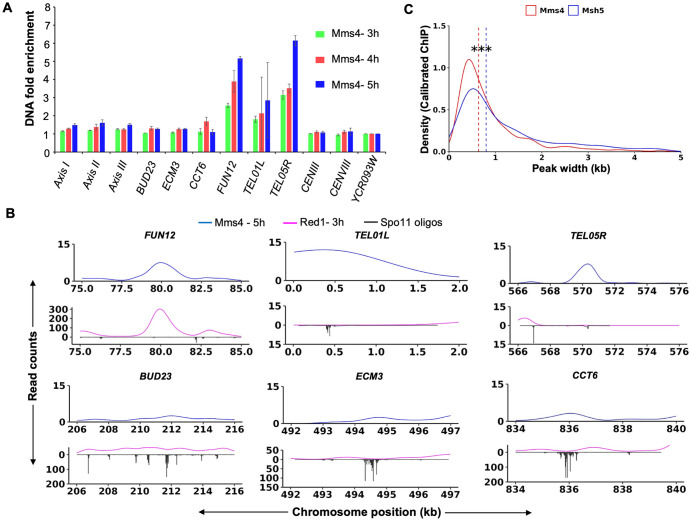
Mms4 shows a trend towards binding weak DSB hotspots. **A)** ChIP-qPCR analysis showing Mms4 enrichment at representative genomic loci, including axis-associated regions (*Axis I*, *Axis II*, and *Axis III)*, DSB hotspots (*BUD23*, *ECM3*, *CCT6*, *FUN12*, *TEL01L*, *TEL05R*), centromeric regions (*CEN III* and *CEN VIII)*, and a DSB coldspot (*YCR093W*). **B)** Mms4 read counts at representative DSB hotspots that show strong (*FUN12*, *TEL01L*, *TEL05R*) and weak (*BUD23*, *ECM3*, and *CCT6*) Mms4 enrichment. **C)** Comparison of Mms4 and Msh5 peak widths at DSB hotspots at 5-hour time point. Red and blue lines indicate the median peak width for Mms4 (0.6 kb) and Msh5 (0.8 kb), respectively. *** indicates p < 0.001 (Wilcoxon rank-sum test).

Analysis of Mms4 ChIP-Seq data showed that Mms4 enrichment was skewed towards weak DSB hotspots compared to Msh5 (Fig 2E and S3 Table). Mms4 and/or Msh5 binding were observed at 718 DSB hotspots (S3 Table). Of these, one third of the DSB hotspots were associated with both Mms4 and Msh5 (S3 Table). However, the Spo11 oligo counts at DSB hotspots specifically enriched for Mms4 were significantly lower than those at DSB hotspots enriched specifically for Msh5 (Fig 2E and S3 Table). This distinction was unlikely due to detection issues, as Mms4 showed a trend towards binding weak DSB hotspots, not strong DSB hotspots, where Mms4 binding would have been easier to detect. It is also in contrast to Msh5, which is preferentially detected at strong DSB hotspots [60]. To further quantify the differences in binding of Mms4 and Msh5, ChIP qPCR analysis was performed on Mms4 ChIP samples at 3,4 and 5h from two independent biological replicates. The analysis initially focused on nine loci, which showed Msh5 binding as reported in [60]. These loci included DSB hotspots (*BUD23*, *ECM3*, and *CCT6*), axis regions (*Axis I*, *Axis II*, and *Axis III*), centromeres (*CENIII* and *CENVIII*), and a DSB coldspot (*YCR093W*). The Mms4 fold enrichment was plotted after dividing by the DSB coldspot value (Fig 3A). At axes and centromere locations analyzed weak Mms4 binding was observed (Fig 3A). Mms4 binding was also weak at the Msh5 enriched DSB hotspots (*BUD23*, *ECM3*, *CCT6*), which was supported by the weak binding of Mms4 observed at these DSB hotspot loci from the ChIP-Seq data as well (Figs 3A and 3B). Therefore, we selected additional DSB hotspots that were specifically enriched for Mms4, based on ChIP-Seq analysis. These included the DSB hotspots: *FUN12*, *TEL01L* and *TEL05R* (Fig 3B). Interestingly, these hotspots exhibited low Spo11 oligo counts (Fig 3B), consistent with our genome-wide analysis, which showed that Mms4-bound hotspots have lower Spo11 oligo counts (Fig 2E and S3 Table). The DNA fold enrichment at these DSB hotspots (*FUN12*, *TEL01L* and *TEL05R*) was enhanced compared to the DSB coldspot *YCR093W* and the *BUD23*, *CCT6* and *ECM3* hotspots (Fig 3A). Together, the genome-wide ChIP-Seq analysis (Fig 2E and S3 Table), supported by locus specific ChIP-qPCR validation suggest that Mms4 shows a trend towards binding weak DSB hotspot regions. We also compared the peak widths of Mms4 and Msh5 at DSB hotspots from the ChIP-Seq data (calibrated 50 bp reads). The median peak width for Mms4 was 0.6 kb (mean 0.9 kb), while for Msh5, the median was 0.8 kb (mean 1.2 kb) (***P < 0.001, Wilcoxon rank-sum test; Fig 3C), suggesting Mms4 peaks were narrower than Msh5 peaks (see discussion).

### Mms4 associates with chromosomes even in the absence of meiotic DSBs

**Fig 4:**
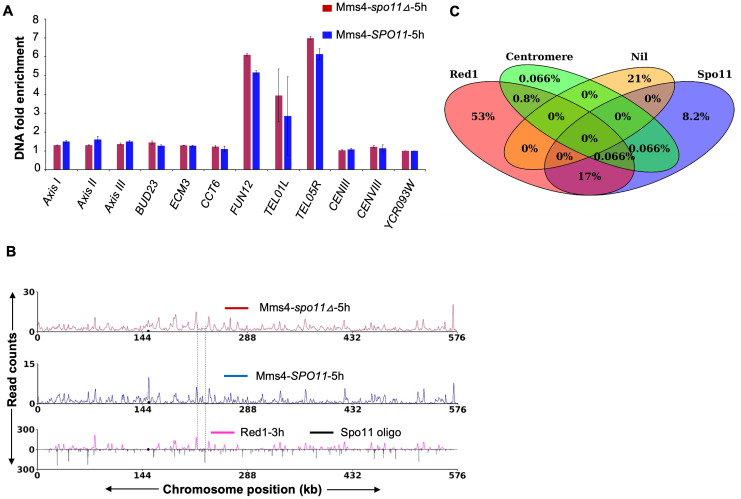
Mms4 associates with meiotic chromosomes in the *spo11Δ* mutant. **A)** ChIP-qPCR analysis showing Mms4 enrichment at representative genomic loci, including axis-associated regions (*Axis I*, *Axis II*, and *Axis III)*, DSB hotspots (*BUD23*, *ECM3*, *CCT6*, *FUN12*, *TEL01L*, *TEL05R*), centromeric regions (*CEN III* and *CEN VIII)*, and a DSB coldspot (*YCR093W*) in a *spo11△* background. **B)** Calibrated ChIP-seq profile showing Mms4-9xMyc binding on chromosome V at 5 hours post meiotic induction in a *spo11△* background. Red1 and Spo11 data are from [19]. Dotted lines indicate Mms4 depletion at DSB hotspots away from the axes in *spo11∆*. **C)** Venn diagram illustrating the overlap of Mms4 binding sites (1509 Mms4 peaks) with DSB hotspots, chromosome axis regions, and centromeres in the *spo11Δ* background, based on a minimum of 1 bp overlap with Mms4 peak coordinates (1509 Mms4 peaks, S2B Table). ‘Nil’ indicates genomic regions not classified under any of these categories.

In *S. cerevisiae* and other organisms, meiotic DSBs are not generated in a *spo11Δ* mutant, resulting in the absence of homologous recombination [4,78]. Consequently, although fragmented axial elements and limited homolog pairing are observed, full length synaptonemal complex formation fails, leading to meiotic arrest or delay at the pachytene stage [15,79,80]. To examine whether meiotic DSB formation stimulates the association of Mms4 with chromosomes during meiosis, we analyzed Mms4 binding in *spo11∆* using ChIP-qPCR, and ChIP-Seq. Mms4 expression in the *spo11Δ* strain was comparable to that in the SK1 wild-type strain, with appearance of the phosphorylated band at 5h (S4A and S4D Fig). The Mms4 ChIP in *spo11∆* showed an expected ~130 kDa Mms4-9xMyc band in the eluate sample (S5A Fig). ChIP-qPCR was performed from two independent biological replicates at representative DSB hotspot loci (*BUD23, ECM3, CCT6, TEL01L, TEL05R, FUN12*), chromosome axis sites (*Axis I*, *Axis II*, *Axis III*), and centromeric regions (*CEN III* and *CEN VIII*). Mms4 binding was similar to wild type at the representative axes, centromere and DSB hotspots (Fig 4A). ChIP-Seq analysis also showed that Mms4 peaks in the *spo11Δ* background were similar to those observed in the wild-type strain (Figs 4B and S2B). However, reduced Mms4 binding was observed in *spo11∆* at some of the DSB hotspots not associated with the axes (Fig 4B). Quantitative analysis of the ChIP-Seq data was used to classify Mms4 peak distribution in the *spo11Δ* background. A Venn diagram was generated to represent the distribution of Mms4-binding sites in *spo11∆*. Mms4 binding at axis sites (53%) and axes associated DSB sites (17%) were similar to wild type (53% and 17% respectively) (Fig 4C). However, the percentage of Mms4 peaks at DSB sites not associated with the axes is reduced to 8.2% from 14% in wild type (Fig 4C and S2B Table) (See discussion). These results suggest Mms4 peaks in *spo11∆* are broadly similar to WT, as most binding occurs at axes. However, Mms4 binding at DSB sites away from the axes is reduced.

### The axis protein Red1 contributes to Mms4 localization

**Fig 5:**
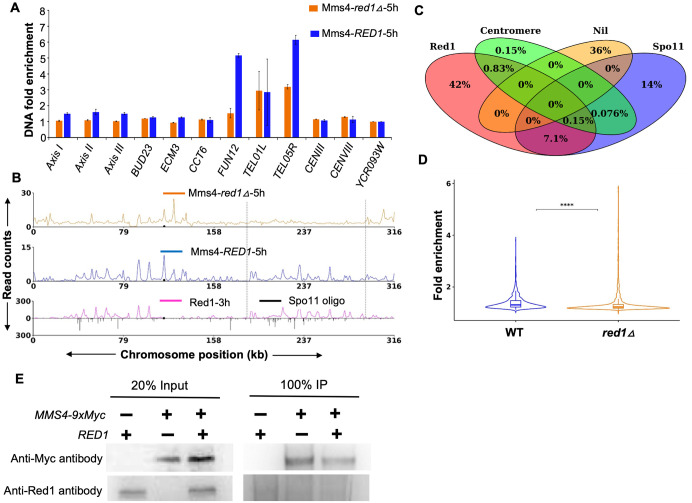
Mms4 binding to meiotic chromosomes is disrupted in the *red1Δ* mutant. **A)** ChIP-qPCR analysis showing Mms4 enrichment at selected genomic loci in *red1∆*. These include axis-associated sites (*Axis I*, *Axis II*, *Axis III)*, DSB hotspots (*BUD23*, *ECM3*, *CCT6*, *FUN12*, *TEL01L*, *TEL05R*), centromeres (*CEN III*, *CEN VIII)*, and a known DSB coldspot (*YCR093W*). **B)** Calibrated ChIP-seq profile of Mms4-9xMyc binding on chromosome III at 5 hours after meiotic induction in a *red1Δ* background. Red1 and Spo11 datasets are from [19] and [21]. Dotted lines indicate Mms4 depletion at axis regions in *red1∆*. **C)** Venn diagram showing the overlap between Mms4 binding sites (1319 Mms4 peaks, S2C Table), DSB hotspots, axis regions, and centromeres, using a threshold of at least 1 bp overlap. Regions outside these categories are grouped under ‘Nil’. **D)** Violin plot showing fold enrichment of Mms4 protein in wild type and *red1∆* compared to the untagged control. **** indicates p < 0.0001 (Wilcoxon rank sum test). **E)** Co-IP analysis to test interaction between Mms4 and Red1 from meiotic cultures (5h post meiotic induction). Controls included inputs and IPs from untagged and *red1Δ* strains. Sample loading was normalized by cell mass, with 20% of input and the entire immunoprecipitated supernatant loaded. Proteins were resolved on 8% SDS-PAGE. Lanes 1 and 4: input and IP for untagged strain; lanes 2 and 5: input and IP for *red1Δ*; lanes 3 and 6: input and IP for Mms4-9xMyc–tagged wild-type strain.

Since Mms4 was mostly associated with the chromosome axes, we investigated the association of Mms4 with meiotic chromosomes in a *red1∆* mutant. The expression pattern of Mms4 in the *red1Δ* strain was comparable to wild-type strain, with appearance of the phosphorylated band at 5h (S4B and S4D Fig). Also, the Mms4 ChIP in *red1∆* showed a band for Mms4-9xMyc (130 kDa) in the eluate fraction (S5A Fig). ChIP-qPCR analysis of Mms4 in the *red1Δ* background (5h) revealed an overall reduction in its binding at all three representative axis-associated regions (*Axis I*, *Axis II*, *Axis III*) and some of the DSB hotspots (*ECM3*, *TEL05R*), including the axis associated *FUN12* hotspot (Fig 5A). However, Mms4 binding at centromeres was unaffected (Fig 5A). Consistent with the ChIP-qPCR results, ChIP-Seq analysis of Mms4 in the *red1Δ* mutant showed reduced association of Mms4 with axes and axes associated DSB hotspots (Figs 5B and S2**C**). Quantitative analysis of the *red1Δ* ChIP-Seq data showed approximately 49.1% of Mms4 peaks overlap with Red1 peaks present in wild-type (including both Red1 and Red1 + Spo11 overlapping Mms4 peaks), which is significantly reduced compared to the 69% overlap observed in the wild-type background (Fig 5C and S2C Table). These results suggest that Red1 contributes to the proper localization or stabilization of Mms4 on meiotic chromosomes. Further, we also observed that the fold enrichment of Mms4 (relative to untagged) was significantly reduced in *red1∆* (1.23 fold) compared to wild type (1.31 fold) (Fig 5D). As seen from the violin plot, the distribution of Mms4 fold enrichment in *red1Δ* was lower compared to wild type (Fig 5D).

We tested whether Mms4 localization at axis regions reflected a direct protein–protein interaction between Mms4 and Red1. We performed co-immunoprecipitation experiments on meiotic extracts (5 hours post-meiotic induction) with C-terminally tagged Mms4-9xMyc and anti-Myc antibodies for pull-downs. In the input fractions, Mms4-9xMyc was present only in the tagged *red1Δ* and wild-type strains (lanes 2 and 3) and absent from the untagged wild type, confirming the genotype controls (Fig 5E). Following immunoprecipitation, Mms4-9xMyc was efficiently recovered from the tagged *red1Δ* and wild-type strains (lanes 5 and 6), but not from the untagged strain (lane 4) (Fig 5E). To test whether Mms4 associates directly with Red1, we probed the samples with a native Red1 antibody. Red1-specific bands were observed in the input fractions of both untagged and tagged wild-type strains (lanes 1 and 3), but were absent in the *red1Δ* strain, as expected (Fig 5E). However, no Red1 bands were detected in the immunoprecipitated fractions of any strain (lanes 4–6). Together, these findings demonstrate that although Red1 contributes to Mms4 localization and both proteins show overlapping binding sites at chromosome axes, direct physical interaction between them could not be detected under our experimental conditions.

### Mms4 binding at DSB hotspots is not enhanced in *msh5∆*

**Fig 6:**
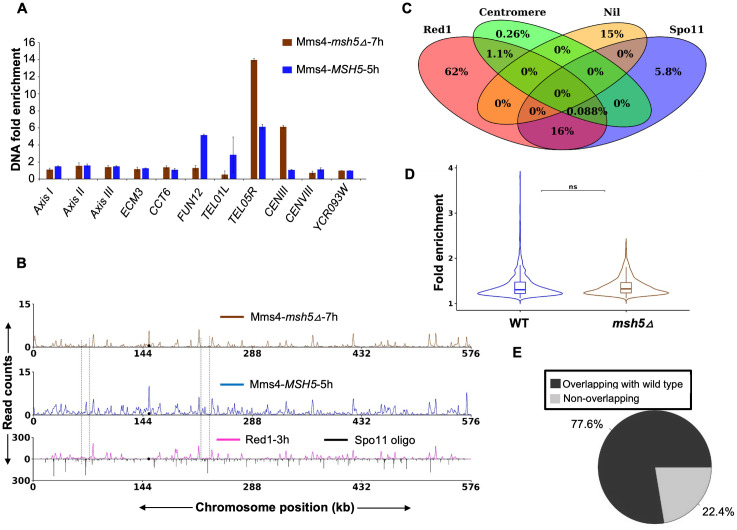
Mms4 binding is not enhanced in the *msh5Δ* mutant. **A)** ChIP-qPCR analysis of Mms4 binding at representative chromosome axes sites (*Axis I*, *Axis II*, *Axis III*), DSB hotspots (*ECM3*, *CCT6*, *FUN12*, *TEL01L*, *TEL05R*), centromeres (*CEN III* and *CEN VIII*), and a DSB coldspot (*YCR093W*). **B)** Calibrated ChIP-seq profile of Mms4-9xMyc binding across chromosome V at 5 hours following meiotic induction in a *msh5Δ* background. Red1 and Spo11 datasets are from [19] and [21]. Dotted lines indicate Mms4 depletion at DSB hotspots away from the axes in *msh5∆*. **C)** Venn diagram depicting the distribution of Mms4 binding sites (1138 Mms4 peaks, S2D Table) in relation to DSB hotspots, chromosome axes, and centromeres, based on at least 1 bp overlap. Peaks not falling into these categories are represented under ‘Nil’. **D)** Violin plot showing fold enrichment of Mms4 protein in wild type and *msh5∆* compared to the untagged control. n.s indicates non significant p value (Wilcoxon rank sum test). **E)** Overlap of Mms4 peaks in wild type versus *msh5∆*.

We sought to determine if Mms4 binding on meiotic chromosomes is enhanced in the absence of the Class I crossover gene *MSH5*. In *msh5Δ* mutants, crossovers and spore viability are reduced by up to 60% and meiotic progression is delayed by 2 hrs [24,81,82]. Mms4 expression in the *msh5Δ* background showed a 2-hour delay, with appearance of the phosphorylated band at 7 hours post-meiotic induction (S4C and S4D Figs). Based on these observations, Mms4 ChIP-qPCR was performed at 7 hours post meiotic induction in *msh5∆*, at representative genomic loci. Mms4 ChIP in *msh5∆* at 7h showed pull down of Mms4-9xMyc (~130 kDa) in the eluate fraction (S5B Fig). Mms4 binding was analyzed at DSB hotspots typically enriched for Msh5 (*ECM3*, and *CCT6*) [60], Mms4 (*TEL01L*, *TEL05R*, and *FUN12*), axis-associated regions (*Axis I*, *Axis II*, and *Axis III*) and centromeric loci (*CEN III* and *CEN VIII*). We observed that Mms4 binding was reduced at *FUN12* and *TEL01L*, while either maintained or elevated at the other loci (Fig 6A). To gain a broader view of Mms4 localization during meiosis in the *msh5Δ* background, Mms4 ChIP-Seq was performed at 7h post meiotic induction from two independent biological replicates. Mms4 read counts were normalized against a control ChIP sample derived from untagged *msh5Δ* cells. Mms4 ChIP-seq data in *msh5Δ* background at 7 h time point (S2D Table) were compared with Mms4 ChIP-seq data from wild-type meiotic cells. Although overall Mms4 peaks in the *msh5Δ* background were similar to those observed in the wild-type strain (Figs 6B and S2D), reduced Mms4 binding was observed in *msh5∆* at some of the DSB hotspots not associated with the axes (Fig 6B).

Quantitative analysis of the ChIP-seq data was used to classify Mms4 peak distribution in the *msh5Δ* background. (S2D Table). A Venn diagram was generated to represent the distribution of Mms4-binding sites across DSB hotspots, chromosome axis sites, and centromeres. Peaks not falling within these annotated regions were categorized as “nil.” There was increased association of Mms4 with axis regions (62%) relative to the wild type. Enrichment at axis associated DSB sites (16%) was similar to that of the wild type. However, a reduced association with Spo11 sites (5.8%) that are not axis-associated was observed (Fig 6C). Further, we also observed that the median fold enrichment of Mms4 (relative to untagged) was similar between *msh5∆* (1.32 fold) and wild type (1.31 fold) at DSB hotspots (Fig 6D). These results suggest that loss of Msh5 does not significantly enhance Mms4 binding at DSB hotspots. Further, we assessed the overlap between Mms4 peaks in the *msh5∆* mutant and wild-type samples. 77.6% of Mms4 peaks in *msh5Δ* at 7 h overlapped with Mms4 peaks in the wild-type background (Fig 6E). Of the 22% non-overlapping Mms4 peaks, 36% showed overlap with Msh5 peaks mostly at axes sites (S4 Table). These results indicate that although no enrichment in Mms4 binding at DSB hotspots is observed in *msh5∆* compared to wild type, there is increased Mms4 association with axis regions at the expense of non-axes associated DSB hotspots (see discussion).

### Mms4 binding is not affected by genomic heterozygosity

**Fig 7:**
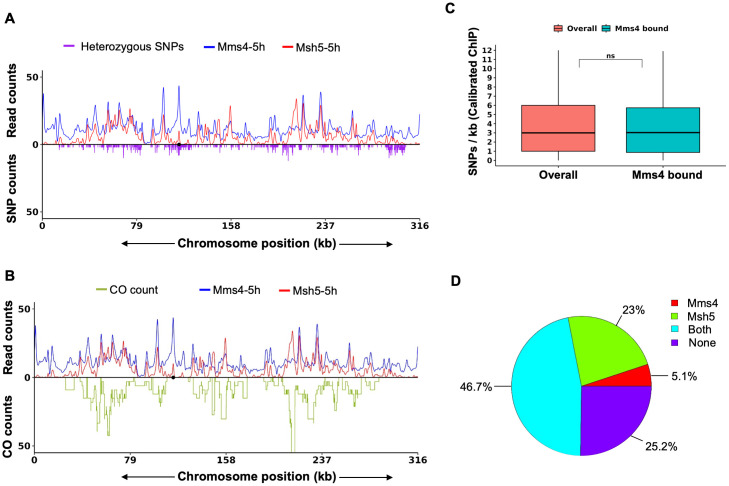
Mms4 binding is independent of SNP density. **A,B**) Representative binding profiles of Mms4 and Msh5 [61] in the S288c-sp/YJM789 hybrid strain on Chromosome III, alongside the corresponding **A)** heterozygous SNP density and **B)** crossover frequency plot. This visualization illustrates the relationship between Mms4 binding and SNP/crossover distribution compared to Msh5. For visualization of the heterozygous SNP counts, the S288c genome was partitioned into 100 bp bins and the number of SNPs were counted in those bins and multiplied by 2. Crossover data was obtained from 66 tetrads of the S288c/YJM789 hybrid [87]. Crossover counts per base were calculated for the S288c genome, and these counts were divided by 66 to get the crossover counts per tetrad for the entire S288c genome. These values were multiplied by 200 for visualization. Wild-type Msh5 binding data were taken from [61] and Msh5 read counts were divided by 4 for visualization. **C)** Box plot comparing genome-wide SNP density (per kb) with the SNP density within Mms4 peaks. Outliers were excluded to enhance visualization. n.s indicates non significant p value (Wilcoxon rank sum test). **D)** Pie chart showing the distribution of crossover sites occupied by Mms4 and Msh5, as a percentage of total crossover sites.

The S288c-sp/YJM789 hybrid is a heterozygous, rapidly sporulating *S. cerevisiae* strain generated by mating *S288c-sp,* which contains sporulation enhancing QTLs (*RME1*, *TAO3*, *MKT1*) derived from *SK1*, with *YJM789* [83,61]. Previous analysis of Msh5 binding in the *S288c-sp/YJM789* hybrid, showed a reduction in Msh5 binding at regions of high heterozygosity [61]. These results were consistent with crossover inhibition at regions with high sequence divergence [84–86]. We tested whether Mms4 binding, which contributes to Class II crossovers is similarly affected by heterozygosity. Mms4 was tagged with 9xMyc independently in *S288c-sp* and *YJM789*, followed by mating of the transformed strains to generate the S288c-sp/YJM789 hybrid with Mms4-9xMyc tag. To directly compare the Mms4 and Msh5 binding profiles in the *S288c-sp/YJM789* hybrid, where Msh5 ChIP was performed at 5 hours, we performed Mms4 ChIP-qPCR and ChIP-Seq at the 5-hour time point as well. ChIP-qPCR analysis of Mms4 in S288c-sp/YJM789 at the DSB hotspots (*FUN12*, *TEL01F*, *TEL05R*, *CCT6*) showed specific enrichment of Mms4 relative to the DSB cold spot (*YCR093W*) (S6 Fig). We analysed Mms4 binding sites (two independent biological replicates at 5h time point) in the S288c-sp/YJM789 hybrid (S5 Table) along with the distribution of heterozygous SNP positions and Msh5 binding profile (Figs 7A and S2E, [61]. Quantitative analysis of the SNP density with Mms4 binding showed SNP density at Mms4 bound sites was similar to the genome wide average (*p*-value = ns, Wilcoxon rank-sum test). The genome-wide mean and median SNP densities were 4.05 SNPs/kb and 3 SNPs/kb, respectively, while Mms4-bound sites showed a comparable mean of 4.14 SNPs/kb and median of 3.26 SNPs/kb (Fig 7C). These results suggest that Mms4 binding is not significantly affected by heterozygosity, unlike Msh5 where SNP density was significantly lower in Msh5 bound regions [61].

To examine the spatial relationship between crossover frequency and the binding profiles of Mms4 and Msh5, we analyzed ChIP-Seq data of Mms4 and Msh5 [61] in conjunction with crossover distribution data from 66 tetrads [87] in the hybrid background. Genome wide visualization revealed a clear overlap between Msh5 and Mms4 peaks and crossover frequency (Figs 7B and S2F). Many of these crossover sites exhibited strong enrichment for both proteins, suggesting their co-occupancy at recombination-active sites. This qualitative analysis suggests that most crossover events are associated with the binding of Msh5 and Mms4, indicating a coordinated role for these proteins during meiotic recombination. Quantitative analysis showed that nearly half of the crossover sites (46.7%) were bound by Mms4 and Msh5, suggesting a strong correlation between crossover activity and the co-localization of these proteins (Fig 7D). Additionally, 23% of the crossover sites were Msh5 associated, while only 5.1% were Mms4 associated. This pattern is consistent with Msh5 generating up to 70% of meiotic crossovers, whereas Mms4 appears to be associated to a smaller subset of sites involving weaker DSB hotspots. 25.2% of crossover sites did not show detectable binding of either protein. These regions may represent sites processed through alternative recombination pathways, or involve transient Mms4 interactions that could not be captured in ChIP-Seq.

## Discussion

The Mms4-Mus81 complex plays a significant role in the repair of mitotic DNA double strand breaks [88,89]. It also contributes up to 30% of crossovers (Class II) in *S. cerevisiae* from the repair of meiotic DNA double strand breaks [41]. Given that there is no information on Mms4 localization on meiotic chromosomes, these data provide valuable insights into the meiotic chromatin landscape accessible to Mms4–Mus81. The ChIP assay detects total Mms4 and therefore does not distinguish between phosphorylated (active) and unphosphorylated (inactive) forms, as the antibodies used recognize all Mms4 species. The aim was to capture the overall chromatin-bound pool, including both active and pre-active states, to identify potential preparatory association sites that may precede catalytic activation. Our results show that Mms4 binds to DSBs, axes, and centromeres on meiotic chromosomes, similar to Msh5. However, comparison of Mms4 and Msh5 binding reveals several differences in the association of the two crossover proteins on meiotic chromosomes: a) Mms4 shows a trend of associating with weak DSB hotspots unlike Msh5; b) Mms4 peak width at DSB hotspots is smaller than Msh5; c) Mms4 binding is observed in *spo11∆* mutant while Msh5 binding is significantly reduced in a *spo11∆* mutant; d) Mms4 binding is not affected by heterozygosity unlike Msh5, consistent with Mms4 chromosomal association, even in the absence of meiotic DSBs. These differences suggest that the binding of Class I and II crossover proteins is distinctly regulated. Further, although Red1 facilitates Mms4 binding to meiotic chromosomes, a direct interaction between them could not be observed under our experimental conditions. It should also be noted that a significant fraction of Mms4 still binds to axis regions in a *red1∆* mutant.

**Fig 8:**
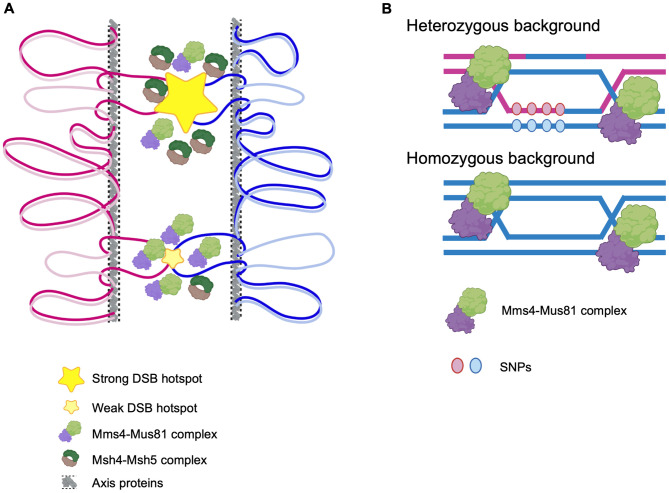
Model for the chromosomal association of the Mms4–Mus81 complex during meiosis. **A) Mms4 shows a trend towards associating with weak DSB hotspots.** In the loop–axis organization of meiotic chromosomes, axis proteins (Red1/Hop1) define the axis, while chromatin loops are tethered to this region for DSB formation and repair. DSBs can be classified as strong sites, with a high probability of break formation, and weak sites. Our analysis suggests that while Mms4–Mus81 may associate with all DSBs, it shows preferential enrichment at weak DSBs compared to strong ones. This pattern contrasts with Msh5, which is predominantly enriched at strong DSB hotspots (see Discussion). **B) Chromosomal association of Mms4 is independent of SNP density.** During meiotic recombination, the Mms4–Mus81 complex resolves joint molecule intermediates, including Holliday junctions, into crossovers or noncrossovers. Our results suggest no significant difference in Mms4 association between heterozygous regions (that generate mismatches during strand invasion) and homozygous contexts. This likely reflects the absence of a role for the Mms4-Mus81 complex in stabilizing strand invasion intermediates or that its binding during meiosis is not dependent on the presence of recombination intermediates that may contain mismatches. In contrast, Msh5 binding is sensitive to mismatch density due to its role in stabilizing strand invasion intermediates. Fig 8A created in BioRender. https://BioRender.com/jh184cj. Fig 8B created in BioRender. https://BioRender.com/h2x55ug.

While most DSB hotspots are associated with both Msh5 and Mms4, there is a trend towards binding slightly stronger DSB hotspots for Msh5 compared to Mms4 (Fig 8A). This suggests that Mms4-Mus81 may function as an alternative or backup pathway at sites that are less efficiently stabilized by ZMM proteins. Our results suggest Mms4 is preferentially associated with weaker DSB hotspots that may be resolved into either crossovers or non-crossovers. Mms4 binding at the strong DSB hotspots (*BUD23*, *ECM3*, *CCT6*) which show Msh5 binding was weak. Strong Mms4 binding was observed at DSB hotspots, such as *FUN12*, *TEL01L*, and *TEL05R*, which have low Spo11 oligo counts (Fig 3A and 3B). Spo11 oligo counts at Mms4 bound DSB hotspots are lower than Spo11 oligo counts at Msh5 bound DSB hotspots (Fig 2E). These differences in Mms4 and Msh5 localization at DSB hotspots are unlikely to result from detection issues as Mms4 shows a trend for binding weak DSB hotspots than strong DSB hotspots. One possibility is that the presence of both Class I and Class II crossover proteins may help to more efficiently process DSBs that are continuously made during meiosis, with the initial breaks (made in early replicating regions) being preferentially bound by Msh5 and the breaks made later (late replicating regions, e.g., telomeres) being bound mostly by Mms4. We also observed that the Mms4 peak width at DSB hotspots was significantly smaller than the Msh5 peak width (Fig 3C). Since Mms4 associates with chromosomes even in the absence of meiotic DSBs, the peak width likely reflects the regional extent of protein association on the chromosome and not necessarily a measure of binding to Holliday junction/recombination intermediates. The difference in peak widths is nevertheless consistent with prior studies indicating that the two proteins associate with Holliday junction substrates differently, with Mms4 having an enzymatic role and Msh5 having a structural role in stabilizing the Holliday junction by forming multiple sliding clamps [90–92].

ChIP-Seq analyses demonstrate that the absence of meiotic DSBs does not disrupt the association of Mms4 protein with chromosomes. In addition, Mms4 expression remains unaffected in *spo11∆* (S4A Fig). The persistence of Mms4 chromosomal localization in *spo11∆* suggests that its association is regulated by mechanisms independent of meiotic DSB formation, potentially through features associated with chromosomal axes. Such a mechanism can explain the strong Mms4 binding observed at *FUN12* and *TEL05* DSB hotspots (Fig 3A), which is reduced in a *red1∆* mutant (Fig 5A). It can also explain why Mms4 binding is specifically reduced at axes independent DSB sites in *spo11∆*. It is also possible that Mms4 associates with replication-associated or other non-meiotic recombination intermediates in *spo11∆*. In contrast to the results with Mms4, previous studies have shown that the chromosomal association of Msh5 during meiosis is significantly enhanced by the formation of Spo11-induced double-strand breaks (DSBs) and recombination intermediates [60].

These observations highlight a fundamental difference in the association of the Mms4 and Msh5 proteins with meiotic chromosomes and suggest that Mms4’s recruitment may not be solely tied to meiotic DSB repair, but is facilitated by axis assembly. Such a mechanism may ensure the availability of Mms4 to process recombination intermediates. Mms4 peaks overlapping with axes and axes associated DSB hotspots were significantly reduced in *red1Δ* background (49%), compared to wild type (69%), along with a significant reduction in Mms4 read density in *red1∆* (Fig 5). These results suggest Red1 plays a role in facilitating or stabilizing the chromosomal association of Mms4 during meiosis. The ChIP-Seq data also show Mms4 binding landscape closely mirrors that of the axis protein Red1. However co-immunoprecipitation assays did not reveal a detectable physical association between the two proteins (Fig 5E). One possible explanation is that their apparent co-localization results from independent recruitment by a common chromatin-associated factor, rather than a direct interaction. Another possibility is that Mms4 and Red1 interact only transiently or in a chromatin-dependent manner, making the association too weak or unstable to withstand protein extraction and immunoprecipitation conditions. It is also conceivable that additional meiotic proteins are required to bridge Mms4 and Red1, such that their interaction is only observable within intact higher-order protein assemblies. Mms4 enrichment at axis sites may also reflect association with regions of topological stress or non-duplex DNA arising from convergent transcription rather than from recombination intermediates alone. This pattern can be interpreted as indicative of a broader chromatin surveillance or readiness function for Mms4-Mus81, consistent with its known ability to recognize a range of branched or stressed DNA structures. These observations suggest that Mms4 binding likely reflects both recombination-dependent and recombination-independent DNA transactions, highlighting the multifaceted role of the Mms4-Mus81 complex in maintaining genome integrity. Also, a plausible model is that the “storage” or pre-activation sites for Mms4-Mus81 are positioned at chromosome axes, as these regions are likely to be in close proximity to recombination intermediates regardless of their location along the chromosome.

### What prevents Mms4 from compensating for loss of Msh5 function?

Mms4 association with meiotic chromosomes was generally lower compared to Msh5. This is expected since Mms4 is part of the minor pathway that resolves joint molecule substrates into crossovers and non-crossovers during meiosis. The other possibility is that the meiotic chromosomes are already saturated with Msh5 at DSB and axes locations, and therefore, we observe reduced Mms4 binding. To test this possibility, we examined Mms4 binding in a *msh5∆* background. Mms4 fold enrichment was not significantly enhanced by the absence of Msh5 (Fig 6D). However, its association with chromosomal axes was enhanced at the expense of DSB hotspots that are not axes associated (Figs 6B and 6C). These results suggest that although Mms4 may be able to access some of the Msh5 associated axis regions in *msh5∆*, it does not act as a backup for Msh5 and relocate to DSB hotspots typically occupied by Msh5 (e.g., *ECM3*, *CCT6*). Genetic observations also support these results, as crossover levels are significantly reduced in the *msh5∆* mutant and are not compensated by Mms4 [32]. The robustness of Mms4’s localization in *msh5∆* indicates that the mechanisms directing its chromosomal binding are stable and not easily perturbed by changes in the recombination landscape. Consistent with this, we also observe from the *spo11∆* mutant, that Mms4 ChIP-Seq signals are not linked to the presence of recombination intermediates (Fig 4). These results are also consistent with the accumulation of DSB repair intermediates in *msh5∆* observed in physical studies, as Mms4 does not process these [28,41]. These results also argue against competition models where Mms4 may be poised at the axes but unable to bind many DSB hotspots due to competition with Msh5. Indeed, we observe that a third of the DSB hotspots are associated with both Mms4 and Msh5 (S3 Table). However, their relative abundance changes at strong and weak DSB hotspots. Although Mms4 binding is not enhanced in *msh5∆*, it may be enhanced in mutants like *sgs1∆* that show enhanced Class II crossovers. This interpretation is further supported by recent observations in *Arabidopsis* showing that the class II pathway does not substantially compensate for loss of Class I crossover machinery [93]. For example, disruption of the MutLγ complex (MLH1–MLH3) results in a strong reduction in Class I crossovers with only limited contribution from MUS81 in resolving ZMM protected recombination intermediate’s [93]. Together, our findings demonstrate that the absence of Mms4 from most DSB sites is not due to exclusion by Msh4/Msh5. However, it remains possible that factors upstream of Msh4/Msh5 (i.e., the ZZS complex [Zip2-Zip4-Spo16] or Mer3) are responsible for that exclusion.

These observations raise an important question regarding the underlying mechanisms that restrict Mms4 from functionally compensating for the loss of Class I crossover proteins (e.g., Msh5). Our findings, together with previous genetic studies, indicate that Mms4-Mus81 contributes only modestly to total crossovers even when ZMM–MutLγ function is disrupted [32,41,33]. The ZMM proteins protect recombination intermediates, that may restrict Mms4-Mus81 access. However, even in the absence of ZMM proteins like Msh5, we do not see enhanced Mms4 binding. One reason may be that helicase–topoisomerase complexes, such as Sgs1–Top3–Rmi1, often dissolve unresolved joint molecules rather than routing them to the class II pathway. Moreover, Mms4-Mus81 activity is temporally constrained, activated only at late stages of prophase I [55]. As a result, its contribution remains quantitatively minor and may not be upregulated in the absence of Class I factors. Unlike Msh5, which promotes interference-sensitive crossovers that are evenly spaced to ensure proper homolog segregation, Mms4–Mus81 generates interference-insensitive crossovers that occur independently of neighboring events [41,32]. While this pathway serves as a safeguard to resolve a small subset of recombination intermediates, its activity must remain limited, as excessive amount of randomly positioned crossovers could lead to aberrant segregation. Unchecked Mms4-Mus81 activity would increase the likelihood of closely spaced crossovers, leading to chromosome mis-segregation. Therefore in systems like *S. cerevisiae*, Mms4–Mus81 is regulated to function as a secondary pathway rather than a major crossover generator, thereby ensuring that recombination remains efficient without compromising the fidelity of meiotic chromosome segregation.

### Mms4 binding is not sensitive to SNP density

Overlap of crossover sites with Mms4 and Msh5 binding sites in the hybrid background provided further insight into their coordinated roles. Msh5, stabilizes both early and late recombination intermediates, such as single end invasions and Holliday junctions [94,90]. Mms4, resolves recombination intermediates, such as Holliday junctions [91]. A substantial proportion (46.7%) of crossover hotspots were co-occupied by both proteins, supporting the model that Msh5 and Mms4 may act together at crossover-designated sites, and provide functional coupling between early and late recombination processes.

The hybrid background also helped us test how heterozygosity affects Mms4 binding. High SNP density inhibits meiotic crossovers among homolog pairs due to the rejection of strand invasion intermediates at regions with high mismatches [84–86]. It is, however, less clear whether this applies to both Class I and Class II crossovers [86,95–97]. In the heterozygous S288c-sp/YJM789 background, Mms4-bound regions showed SNP densities comparable to genome-wide averages (Fig 7C), whereas previous work showed reduced Msh5 binding at highly polymorphic loci in the same hybrid [61]. These observations indicate that, unlike Msh5, Mms4 genomic association is not strongly correlated with local SNP density. These results suggest that proteins like Msh5 which act at early stages of the recombination process are sensitive to homology requirements for binding and stabilizing the strand invasion intermediates. On the other hand, downstream resolution factors like Mms4 may engage recombination intermediates solely based on their conformation and may be buffered against the effects of sequence polymorphism that affect early stage recombination proteins (Fig 8B). It is also possible that the lack of effect of SNP density on Mms4 binding, may simply reflect the fact that Mms4 binding is not dependent on the presence of recombination intermediates (Fig 4). These results highlight distinct differences in how the Class I and Class II crossover proteins interact with a heterozygous genome.

## Supporting information

S1 Fig A) Spore viability analysis of tetrads dissected from the *MMS4-9xMyc* tagged strain and the untagged wild-type (WT) SK1 strain. n = number of tetrads dissected from two independent sporulated cultures. B) Sporulation efficiency of the *MMS4-9xMyc* strain assessed alongside the wild-type strain. Meiotic progression was monitored by DAPI staining of nuclei from two independent replicates.
(PDF)

S2 Fig A) Calibrated ChIP-Seq profile showing Mms4-9xMyc binding across all sixteen chromosomes at 3,4 and 5 hours post meiotic induction. Red1 and Spo11 data are from [19] and [21], respectively. The black circle marks the centromere. B) Calibrated ChIP-Seq profile showing Mms4-9xMyc binding for all sixteen chromosomes at 5 hours post meiotic induction in a *spo11△* background. Red1 and Spo11 data are from [19] and [21], respectively. The black circle marks the centromere. C) Calibrated ChIP-Seq profile showing Mms4-9xMyc binding for all sixteen chromosomes at 5 hours post meiotic induction in a *red1△* background. Red1 and Spo11 data are from [19] and [21] respectively. The black circle marks the centromere. D) Calibrated ChIP-Seq profile showing Mms4-9xMyc binding for all sixteen chromosomes at 5 hours post meiotic induction in a *msh5△* background. Red1 and Spo11 data are from [19] and [21] respectively. The black circle marks the centromere. E) Binding profiles of Mms4 and Msh5 in the S288c-sp/YJM789 hybrid strain for all sixteen chromosomes alongside the corresponding heterozygous SNP density plot. This visualization illustrates the relationship between Mms4 binding and SNP distribution compared to Msh5. For visualization of the heterozygous SNP counts, the S288c genome was partitioned into 100 bp bins and the number of SNPs were counted in those bins and multiplied by 2. Wild type Msh5 binding data were taken from [61] and Msh5 read counts were divided by 4 for visualization. F) Binding profiles of Mms4 and Msh5 in the S288c-sp/YJM789 hybrid strain for all sixteen chromosomes alongside the corresponding crossover frequency plot. This visualization illustrates the relationship between Mms4 binding and crossover distribution compared to Msh5. Crossover data was obtained from 66 tetrads of the S288c/YJM789 hybrid [87]. Crossover counts per base were calculated for the S288c genome, and these counts were divided by 66 to get the crossover counts per tetrad for the entire S288c genome. These values were multiplied by 200 for visualization. Wild-type Msh5 binding data were taken from [61] and Msh5 read counts were divided by 4 for visualization.
(PDF)

S3 Fig A) Locations of DSB hotspots uniquely associated with Mms4 or Msh5. Black circles show the centromere on each chromosome. B) Bar plot representing distance from the centromere for DSB hotspots bound uniquely by Mms4 or Msh5. ns indicates non-significant p value (Wilcoxon-Rank Sum test).
(PDF)

S4 Fig Mms4 expression analysis in mutants. Western blot analysis of Mms4 expression from 0 to 8h post onset of meiosis in A) *spo11△,* B) *red1△* and C) *msh5△*. For comparison, Mms4 expression in the wild-type strain (0-9h) is also shown (D). Pgk1 expression is shown as a control in all of the above blots.
(PDF)

S5 Fig Mms4 ChIP in mutants. Immunoblot analysis of Mms4-9xMyc ChIP using anti-Myc antibody in synchronised meiotic cultures of A) *red1△, spo11△* and B) *msh5△* mutants. A) Lanes 1, 2 and 3 for *red1△* and lanes 4, 5, and 6 for *spo11△* strains indicate lysate before incubation with magnetic beads, lysate after incubation with beads, and eluate fraction. M represents marker. B) Lanes 1, 2 and 3 for *msh5△* strains indicate lysate before incubation with magnetic beads, lysate after incubation with beads, and eluate fraction.
(PDF)

S6 Fig ChIP-qPCR analysis showing Mms4 enrichment at representative DSB hotspots (*FUN12*, *TEL01L*, *TEL05R, CCT6*) with reference to the DSB coldspot (*YCR093W*) in the S288c-sp/YJM789 hybrid at 5h time point. Data are from two independent biological replicates.
(PDF)

S1 Table Strains used in this study. (PDF)

S2 Table A) Mms4 peak locations at 5h time point in SK1 wild type strain. Label indicates overlap of Mms4 peak with Spo11 peaks, Red1 peaks and centromeres. B) Mms4 peak locations at 5h time point in the *spo11∆* mutant. Label indicates overlap of Mms4 peak with Spo11 peaks, Red1 peaks and centromeres. Nil indicates other regions. p value indicates statistical significance of the peaks, q value indicates the adjusted p value using Benjamini Hochberg correction. C) Mms4 peak locations at 5h time point in the *red1∆* mutant. Label indicates overlap of Mms4 peak with Spo11 peaks, Red1 peaks and centromeres. Mms4 peaks with Nil label indicates other regions. p value indicates statistical significance of the peaks, q value indicates the adjusted p value using Benjamini Hochberg correction. D) Mms4 peak locations at 5h time point in the *msh5∆* mutant. Label indicates overlap of Mms4 peak with Spo11 peaks, Red1 peaks and centromeres. Nil label indicates other regions. p value indicates statistical significance of the peaks, q value indicates the adjusted p value using Benjamini Hochberg correction.
(XLSX)

S3 Table DSB hotspots showing overlap with peaks corresponding to either Mms4, Msh5 or both. Table is sorted based on Spo11 oligo hits. Mean and Median Spo11 oligo counts are shown for DSB hotspots associated uniquely with Mms4 or Msh5.
(XLSX)

S4 Table Unique Mms4 peaks in *msh5∆* that overlap with wild type Msh5 peaks. (XLSX)

S5 Table Mms4 peak locations at 5h time point in the S288c-sp/YJM789 wild type strain. p value indicates statistical significance of the peaks, q value indicates the adjusted p value using Benjamini Hochberg correction.
(XLSX)
