# Supplementary material for: Mms4 chromosomal association reveals functional relationships between meiotic crossover pathways in budding yeast
Source: PLoS Genet. 2026 Mar 30;22(3):e1012097. doi: 10.1371/journal.pgen.1012097 (PMC13046247; doi:10.1371/journal.pgen.1012097)
Supplement: S1 Fig — B) Sporulation efficiency of the MMS4-9xMyc strain assessed alongside the wild-type strain. Meiotic progression was monitored by DAPI staining of nuclei from two independent replicates. (PDF) [file pgen.1012097.s001.pdf]

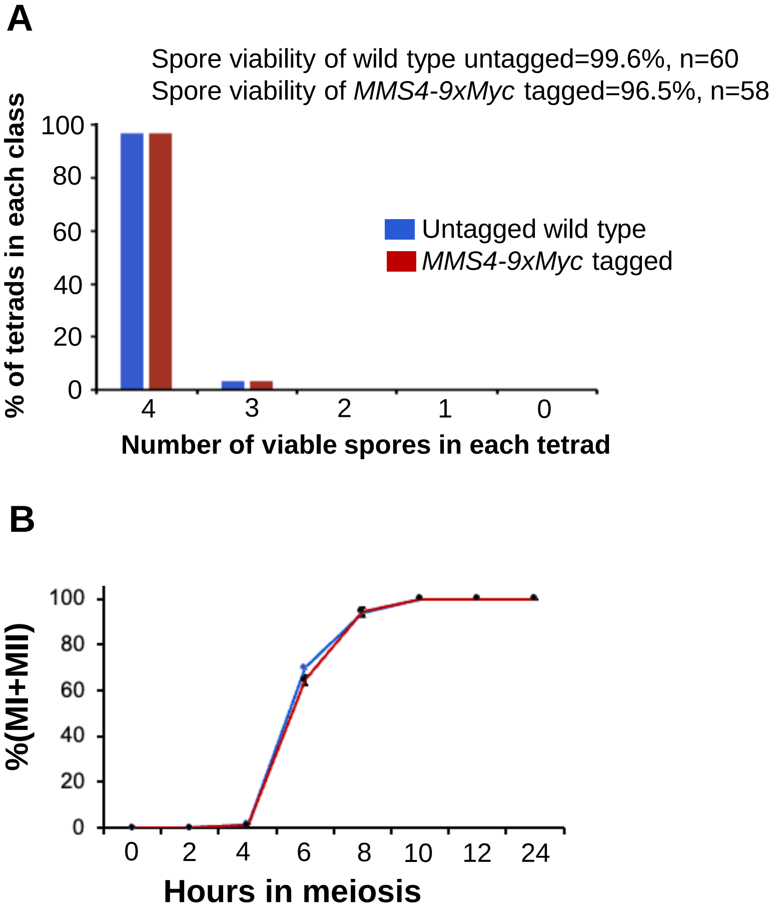

S1 Fig. **A)** Spore viability analysis of tetrads dissected from the *MMS4-9xMyc* tagged strain and the untagged wild-type (WT) SK1 strain. n = number of tetrads dissected from two independent sporulated cultures. **B)** Sporulation efficiency of the *MMS4-9xMyc* strain assessed alongside the wild-type strain. Meiotic progression was monitored by DAPI staining of nuclei from two independent replicates.
