# Supplementary material for: Mms4 chromosomal association reveals functional relationships between meiotic crossover pathways in budding yeast
Source: PLoS Genet. 2026 Mar 30;22(3):e1012097. doi: 10.1371/journal.pgen.1012097 (PMC13046247; doi:10.1371/journal.pgen.1012097)
Supplement: S2 Fig — Red1 and Spo11 data are from [19] and [21], respectively. The black circle marks the centromere. B) Calibrated ChIP-Seq profile showing Mms4-9xMyc binding for all sixteen chromosomes at 5 hours post meiotic induction in a spo11△ background. Red1 and Spo11 data are from [19] and [21], respectively. The black circle marks the centromere. C) Calibrated ChIP-Seq profile showing Mms4-9xMyc binding for all sixteen chromosomes at 5 hours post meiotic induction in a red1△ background. Red1 and Spo11 data are from [19] and [21] respectively. The black circle marks the centromere. D) Calibrated ChIP-Seq profile showing Mms4-9xMyc binding for all sixteen chromosomes at 5 hours post meiotic induction in a msh5△ background. Red1 and Spo11 data are from [19] and [21] respectively. The black circle marks the centromere. E) Binding profiles of Mms4 and Msh5 in the S288c-sp/YJM789 hybrid strain for all sixteen chromosomes alongside the corresponding heterozygous SNP density plot. This visualization illustrates the relationship between Mms4 binding and SNP distribution compared to Msh5. For visualization of the heterozygous SNP counts, the S288c genome was partitioned into 100 bp bins and the number of SNPs were counted in those bins and multiplied by 2. Wild type Msh5 binding data were taken from [61] and Msh5 read counts were divided by 4 for visualization. F) Binding profiles of Mms4 and Msh5 in the S288c-sp/YJM789 hybrid strain for all sixteen chromosomes alongside the corresponding crossover frequency plot. This visualization illustrates the relationship between Mms4 binding and crossover distribution compared to Msh5. Crossover data was obtained from 66 tetrads of the S288c/YJM789 hybrid [87]. Crossover counts per base were calculated for the S288c genome, and these counts were divided by 66 to get the crossover counts per tetrad for the entire S288c genome. These values were multiplied by 200 for visualization. Wild-type Msh5 binding data were taken from [61] and Msh5 read coun [file pgen.1012097.s002.pdf]

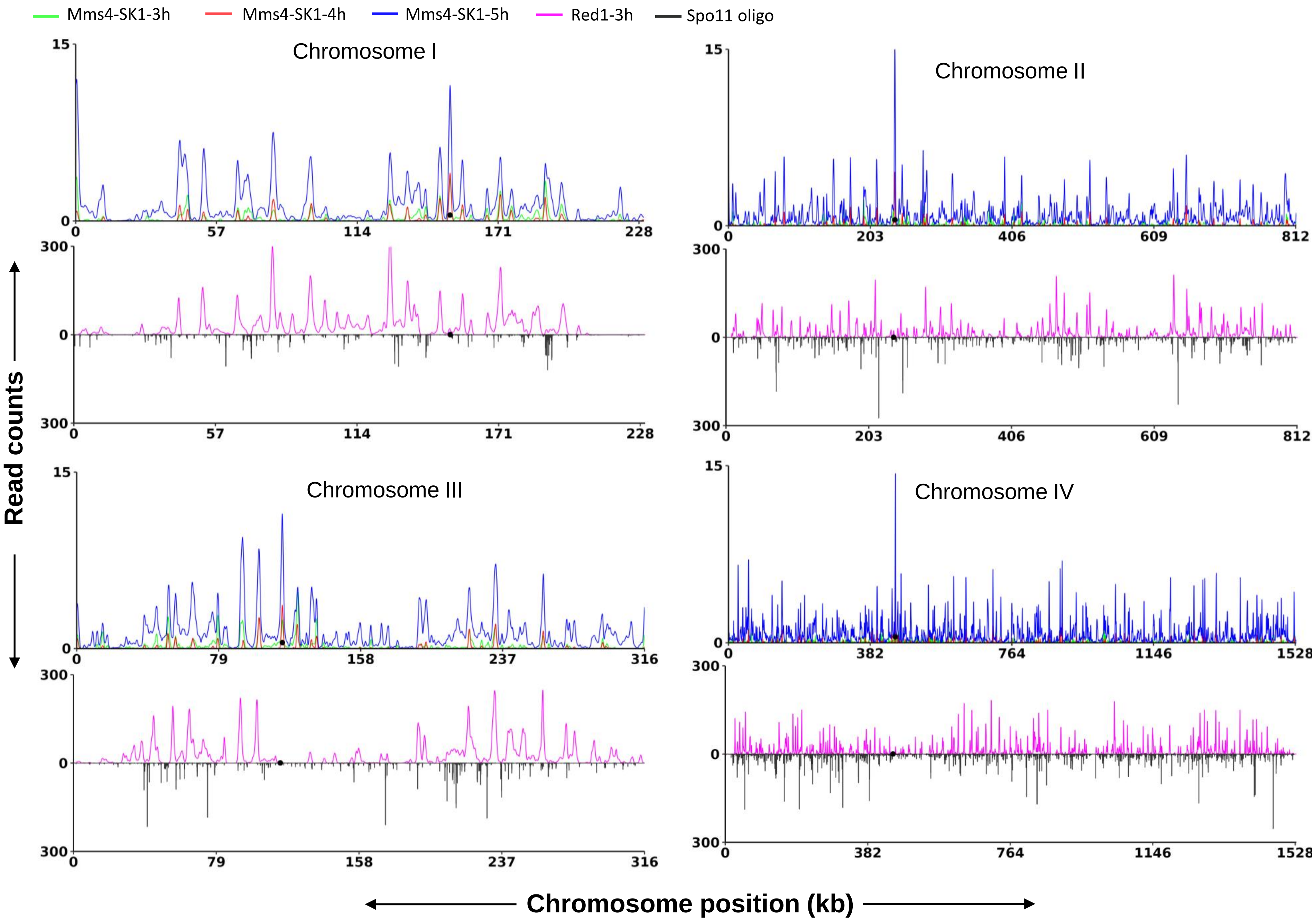

S2 Fig. A) Calibrated ChIP-Seq profile showing Mms4-9xMyc binding across all sixteen chromosomes at 3,4 and 5 hours post meiotic induction. Red1 and Spo11 data are from Sun *et al.,* 2015 and Pan *et al.,* 2011, respectively. The black circle marks the centromere.

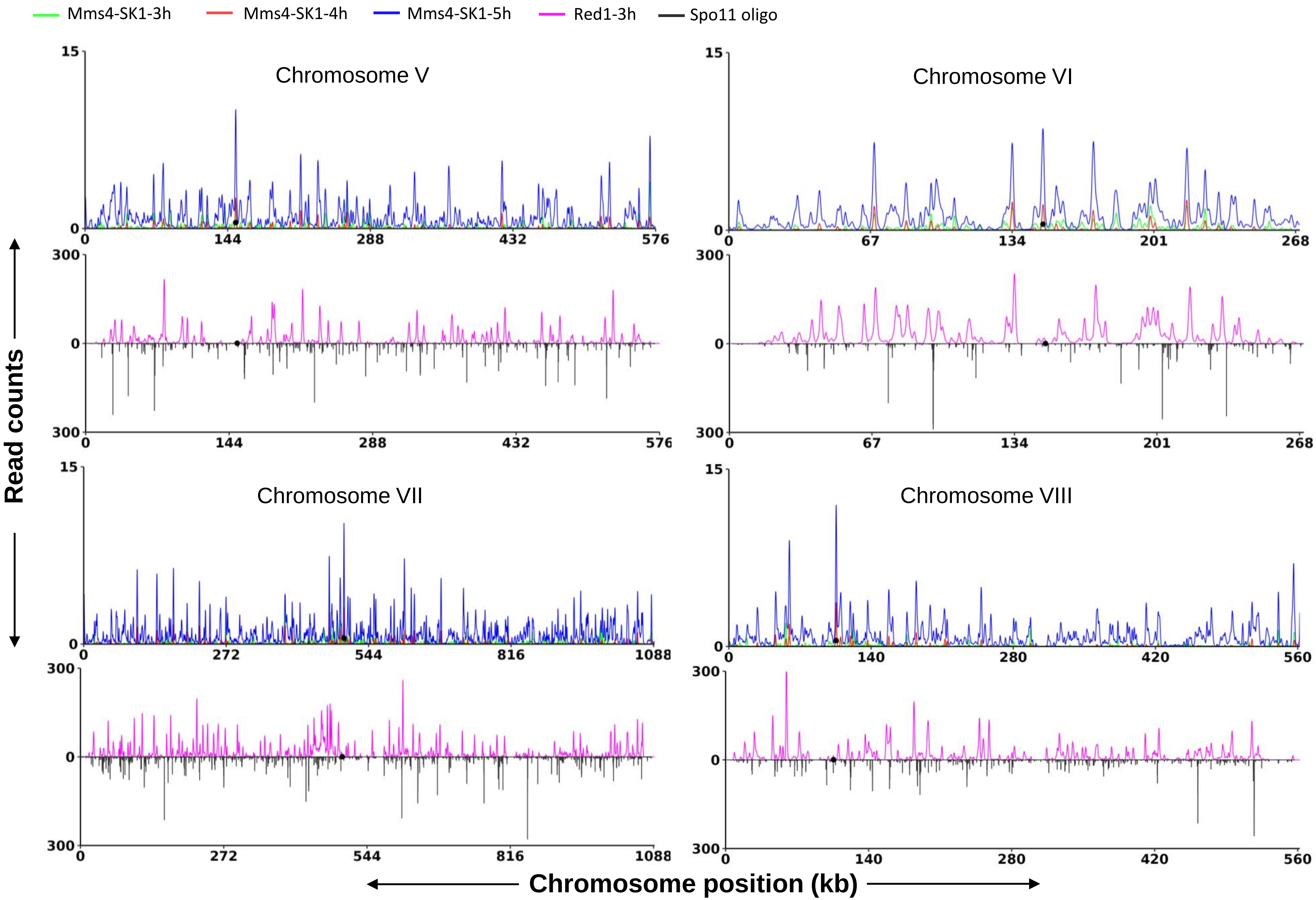

S2 Fig. A) Calibrated ChIP-Seq profile showing Mms4-9xMyc binding across all sixteen chromosomes at 3,4 and 5 hours post meiotic induction. Red1 and Spo11 data are from Sun *et al.,* 2015 and Pan *et al.,* 2011, respectively. The black circle marks the centromere.

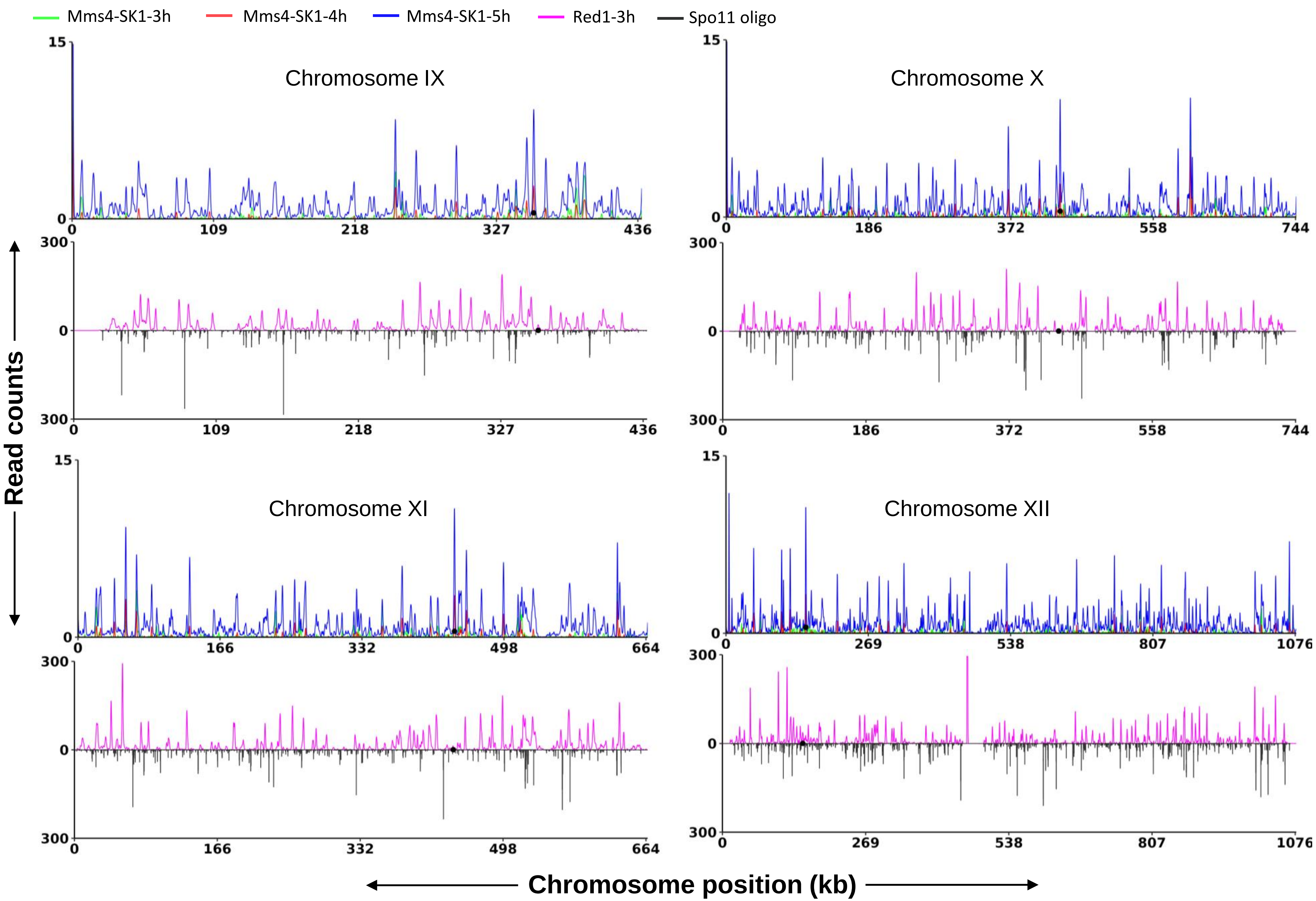

S2 Fig. A) Calibrated ChIP-Seq profile showing Mms4-9xMyc binding across all sixteen chromosomes at 3,4 and 5 hours post meiotic induction. Red1 and Spo11 data are from Sun *et al.,* 2015 and Pan *et al.,* 2011, respectively. The black circle marks the centromere.

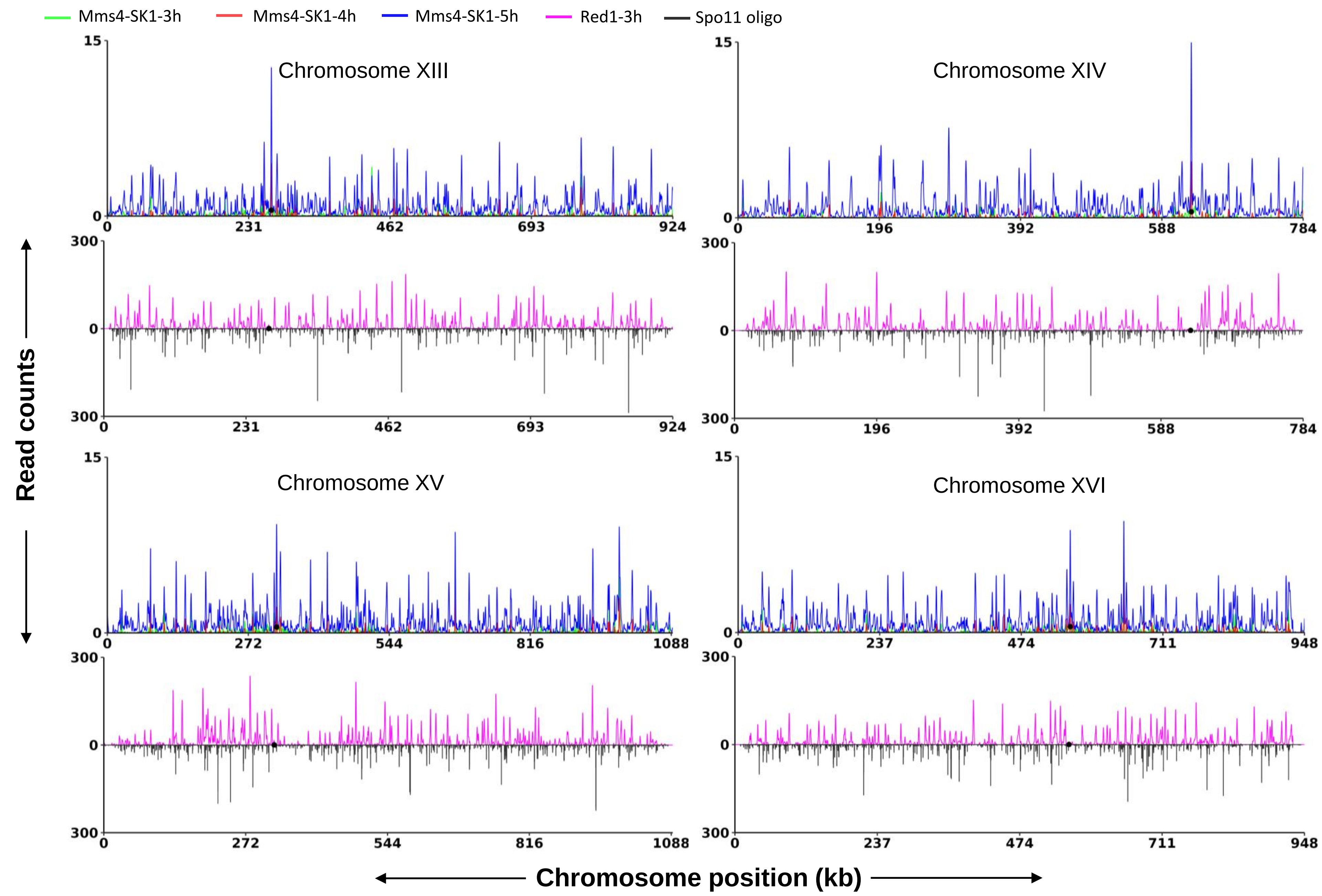

S2 Fig. A) Calibrated ChIP-Seq profile showing Mms4-9xMyc binding across all sixteen chromosomes at 3,4 and 5 hours post meiotic induction. Red1 and Spo11 data are from Sun *et al.,* 2015 and Pan *et al.,* 2011, respectively. The black circle marks the centromere.

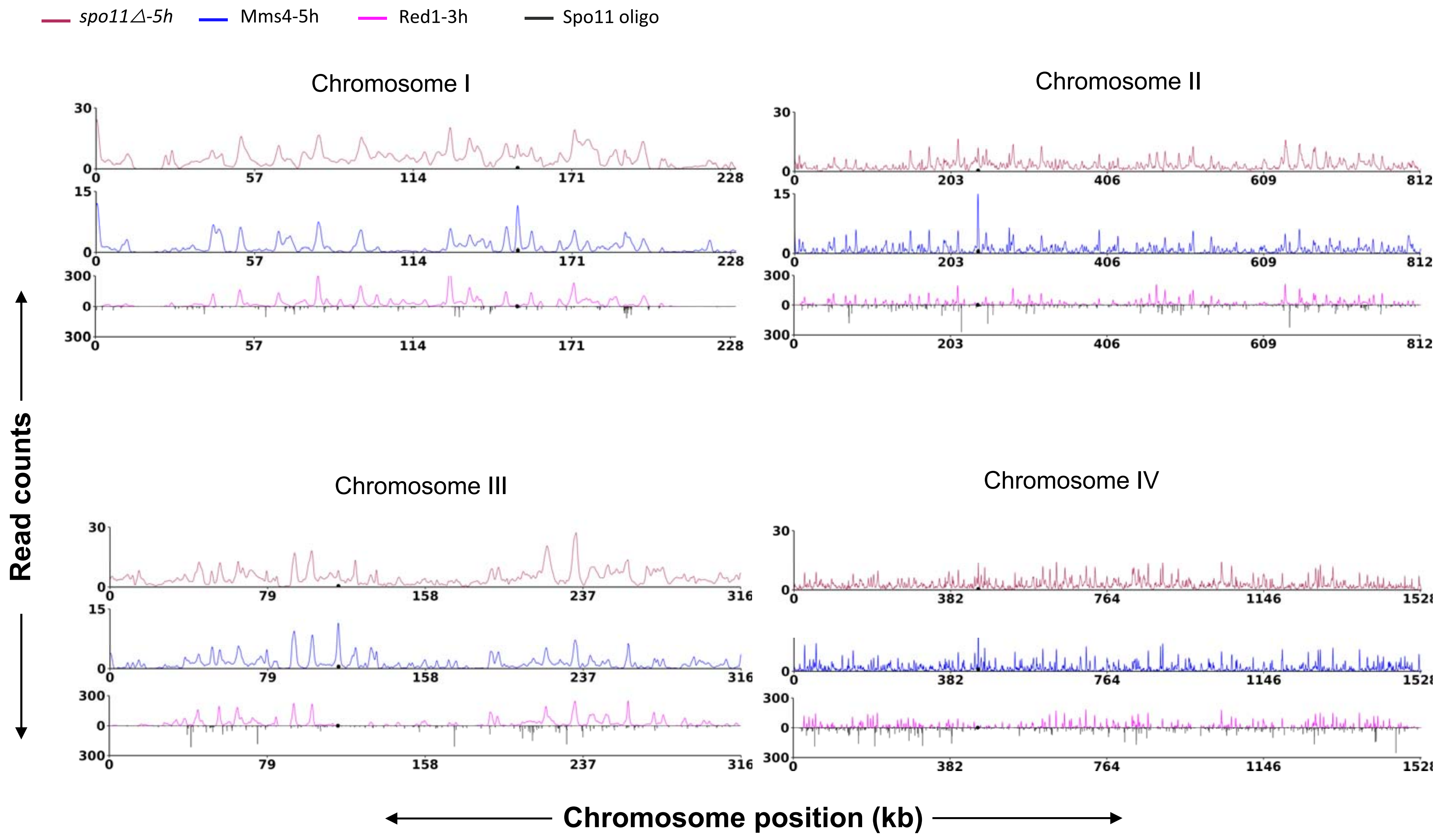

S2 Fig. B) Calibrated ChIP-Seq profile showing Mms4-9xMyc binding for all sixteen chromosomes at 5 hours post meiotic induction in a *spo11Δ* background. Red1 and Spo11 data are from Sun *et al.,* 2015 and Pan *et al.,* 2011, respectively.
The black circle marks the centromere.

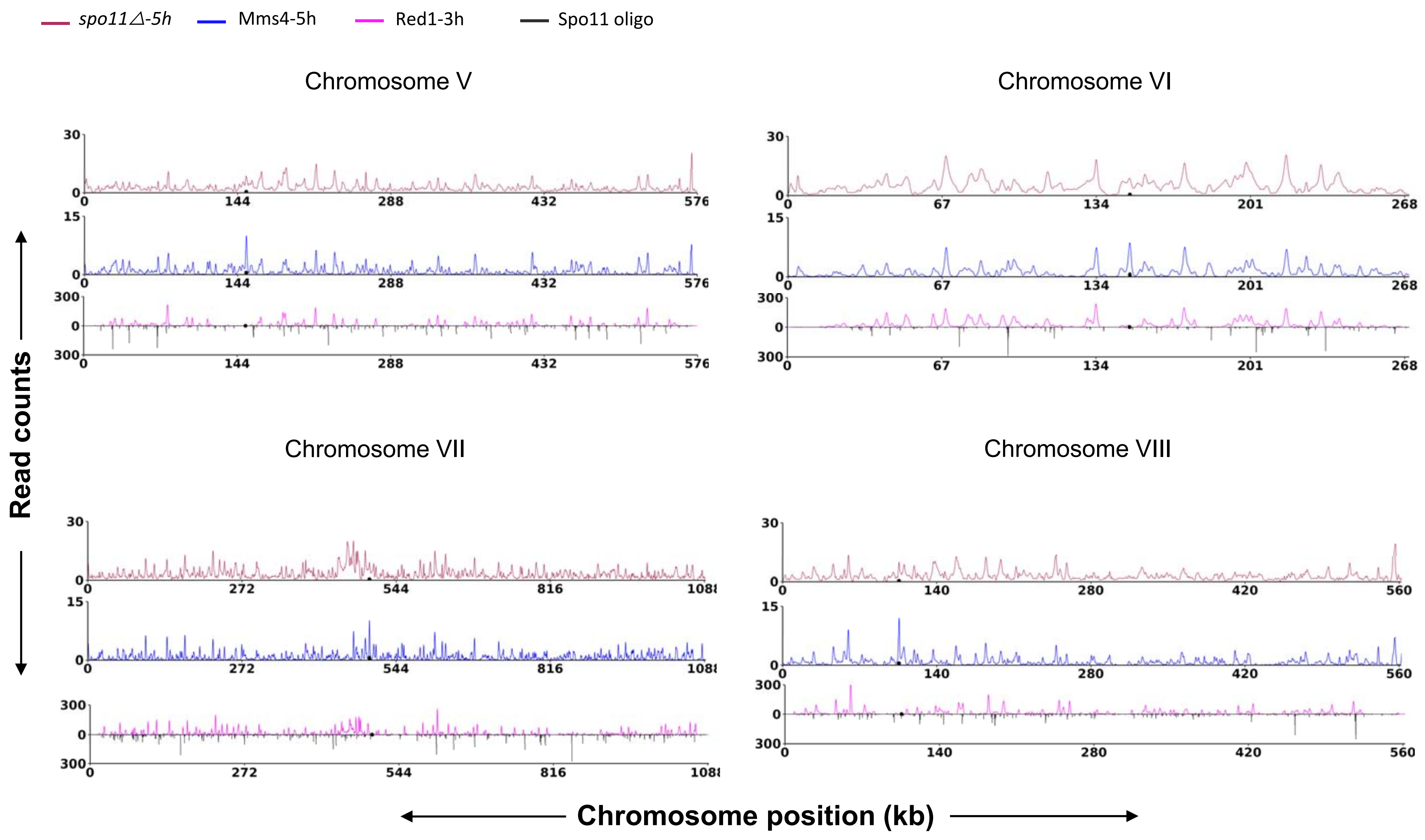

S2 Fig. B) Calibrated ChIP-Seq profile showing Mms4-9xMyc binding for all sixteen chromosomes at 5 hours post meiotic induction in a *spo11Δ* background. Red1 and Spo11 data are from Sun *et al.,* 2015 and Pan *et al.,* 2011, respectively.
The black circle marks the centromere.

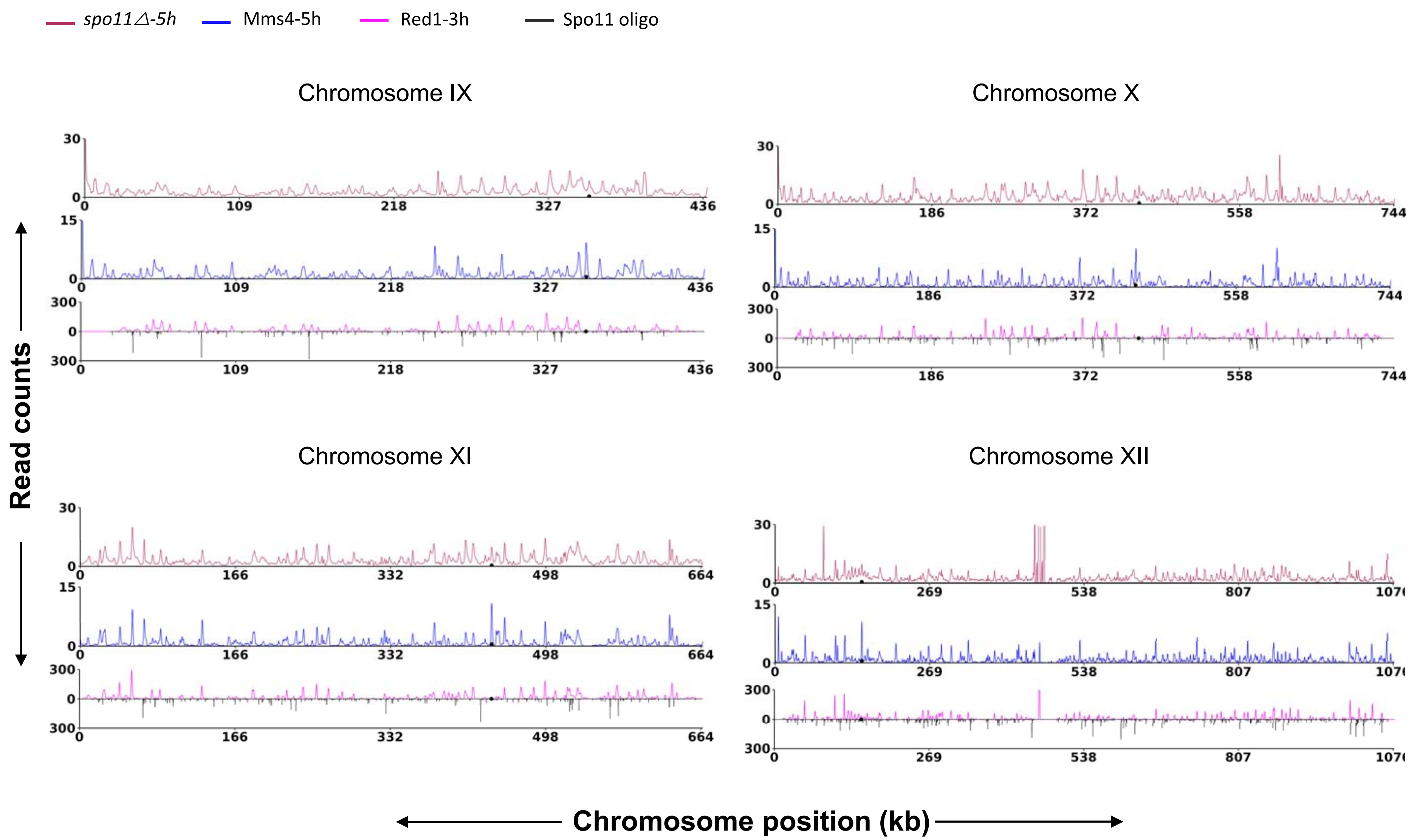

S2 Fig. B) Calibrated ChIP-Seq profile showing Mms4-9xMyc binding for all sixteen chromosomes at 5 hours post meiotic induction in a *spo11Δ* background. Red1 and Spo11 data are from Sun *et al.,* 2015 and Pan *et al.,* 2011, respectively.
The black circle marks the centromere.

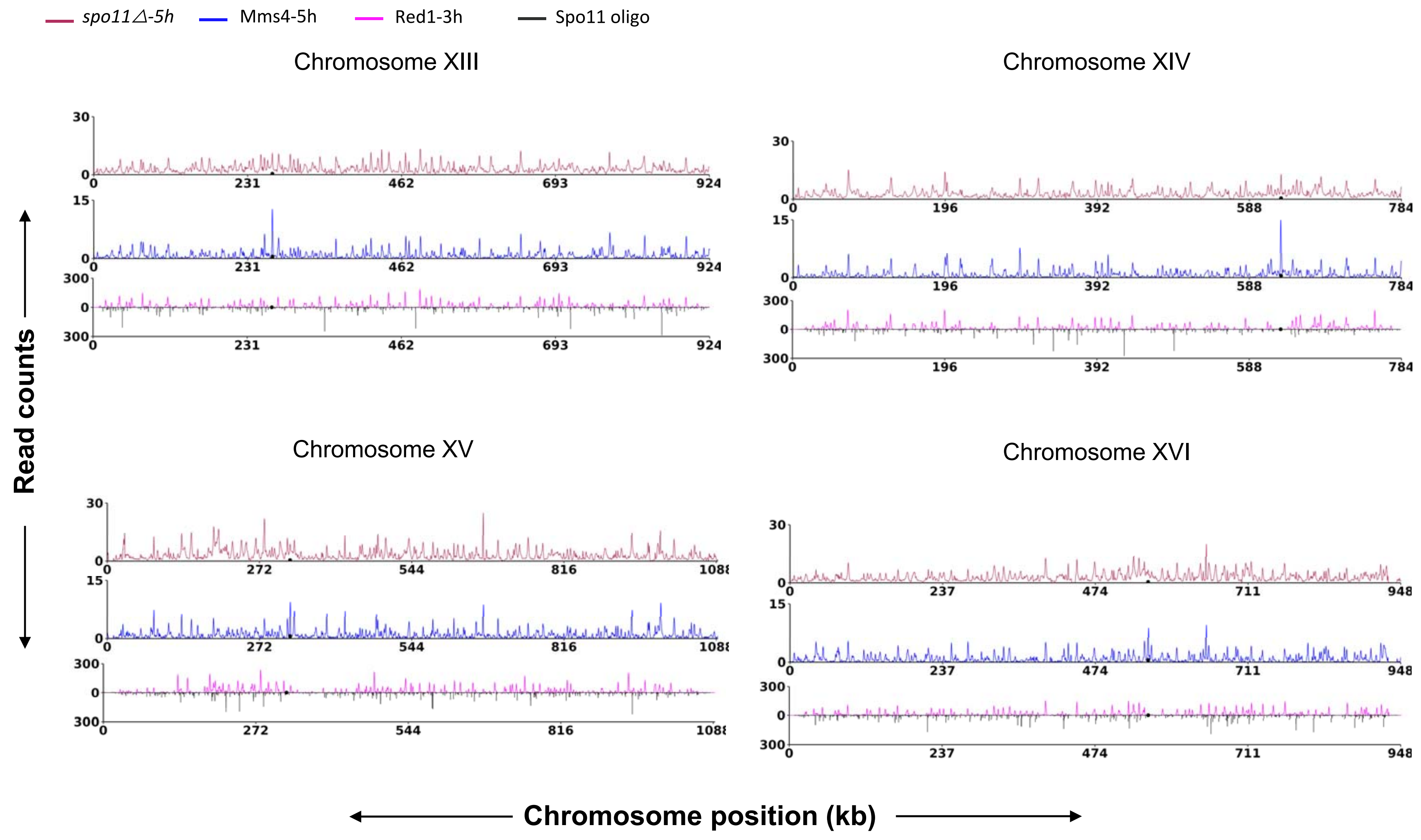

S2 Fig. B) Calibrated ChIP-Seq profile showing Mms4-9xMyc binding for all sixteen chromosomes at 5 hours post meiotic induction in a *spo11Δ* background. Red1 and Spo11 data are from Sun *et al.*, 2015 and Pan *et al.*, 2011, respectively.
The black circle marks the centromere.

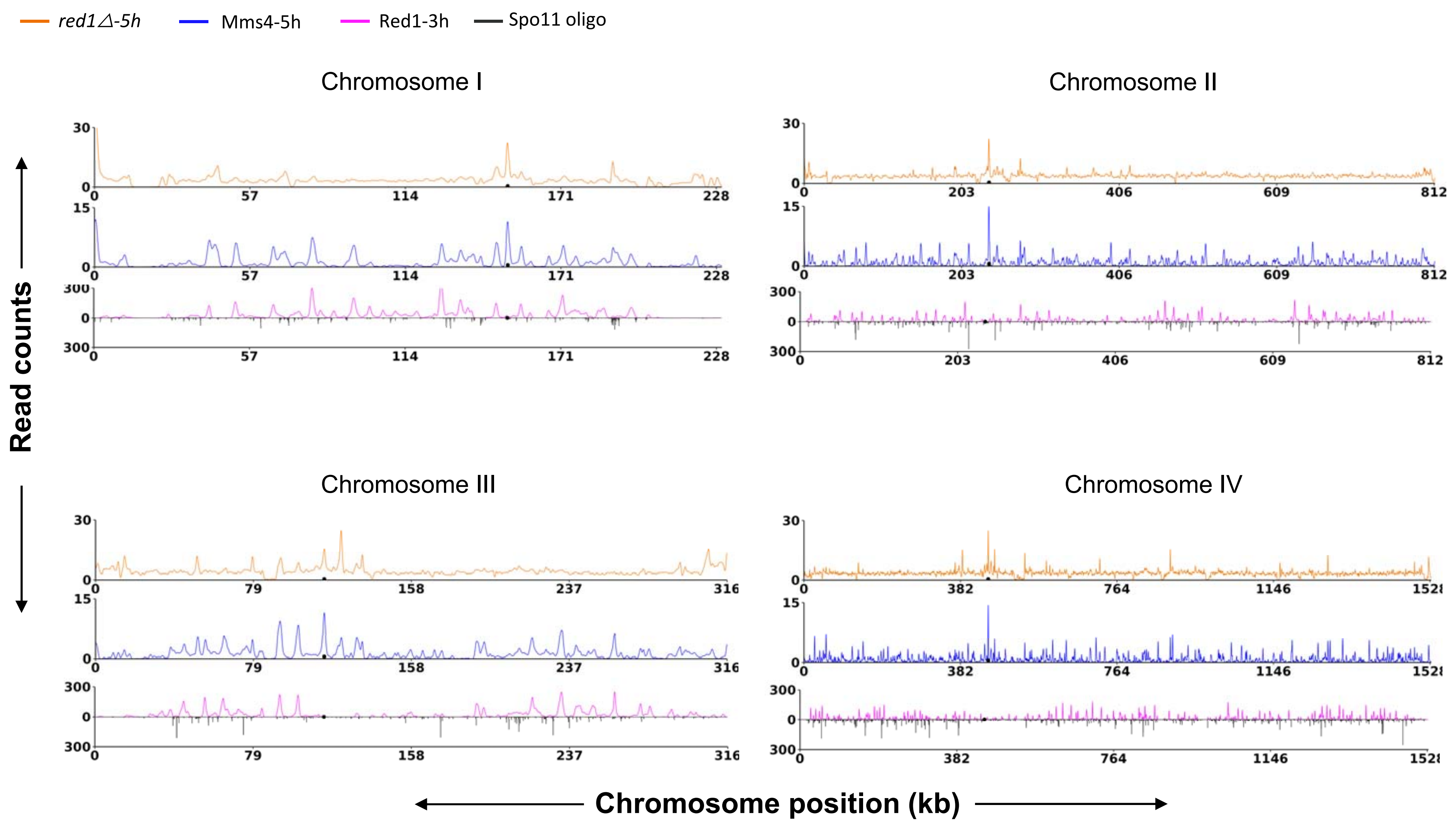

S2 Fig. C) Calibrated ChIP-Seq profile showing Mms4-9xMyc binding for all sixteen chromosomes at 5 hours post meiotic induction in a *red1Δ* background. Red1 and Spo11 data are from Sun *et al.*, 2015 and Pan *et al.*, 2011 respectively.
The black circle marks the centromere.

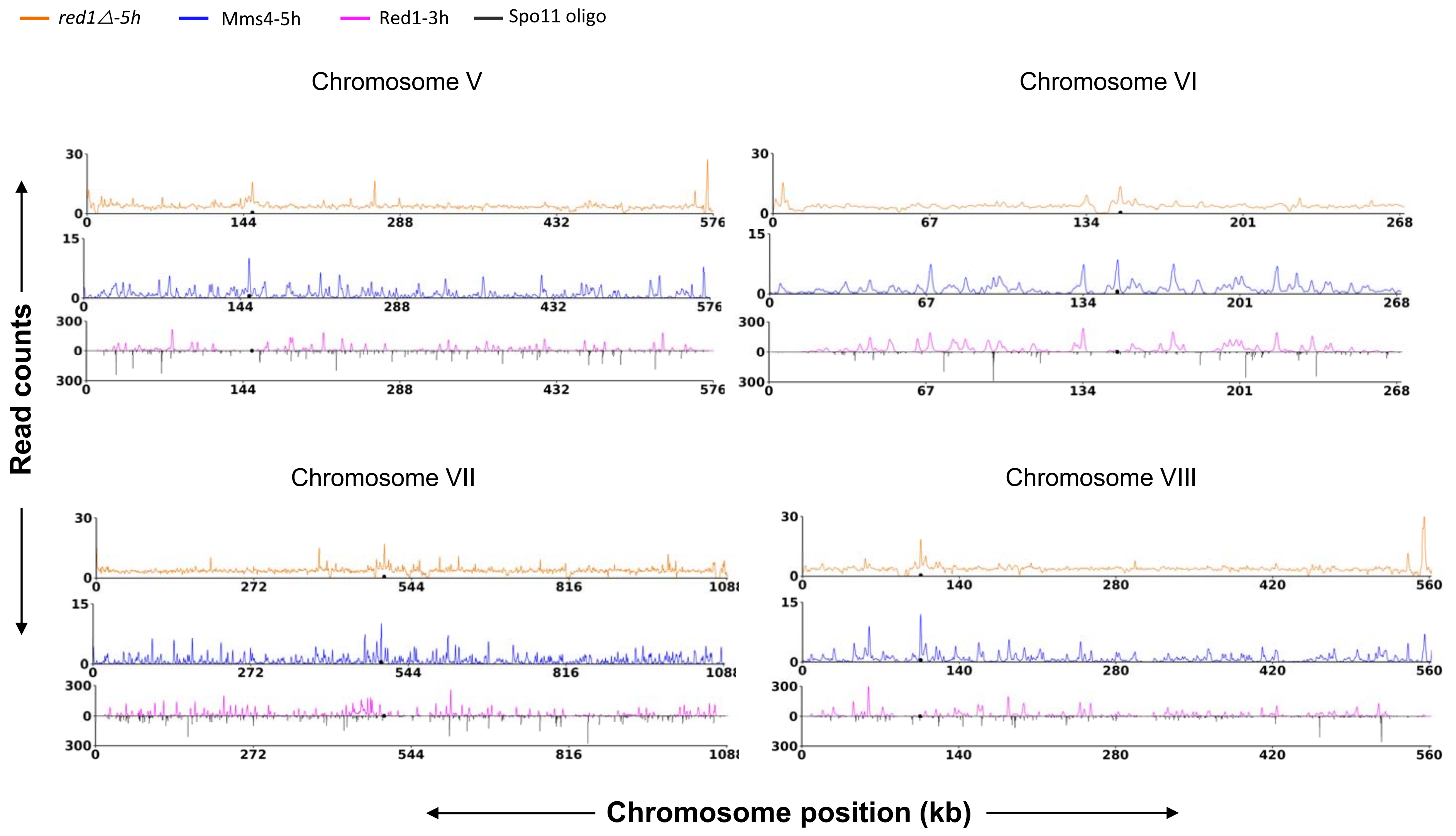

S2 Fig. C) Calibrated ChIP-Seq profile showing Mms4-9xMyc binding for all sixteen chromosomes at 5 hours post meiotic induction in a *red1Δ* background. Red1 and Spo11 data are from Sun *et al.*, 2015 and Pan *et al.*, 2011 respectively.
The black circle marks the centromere.

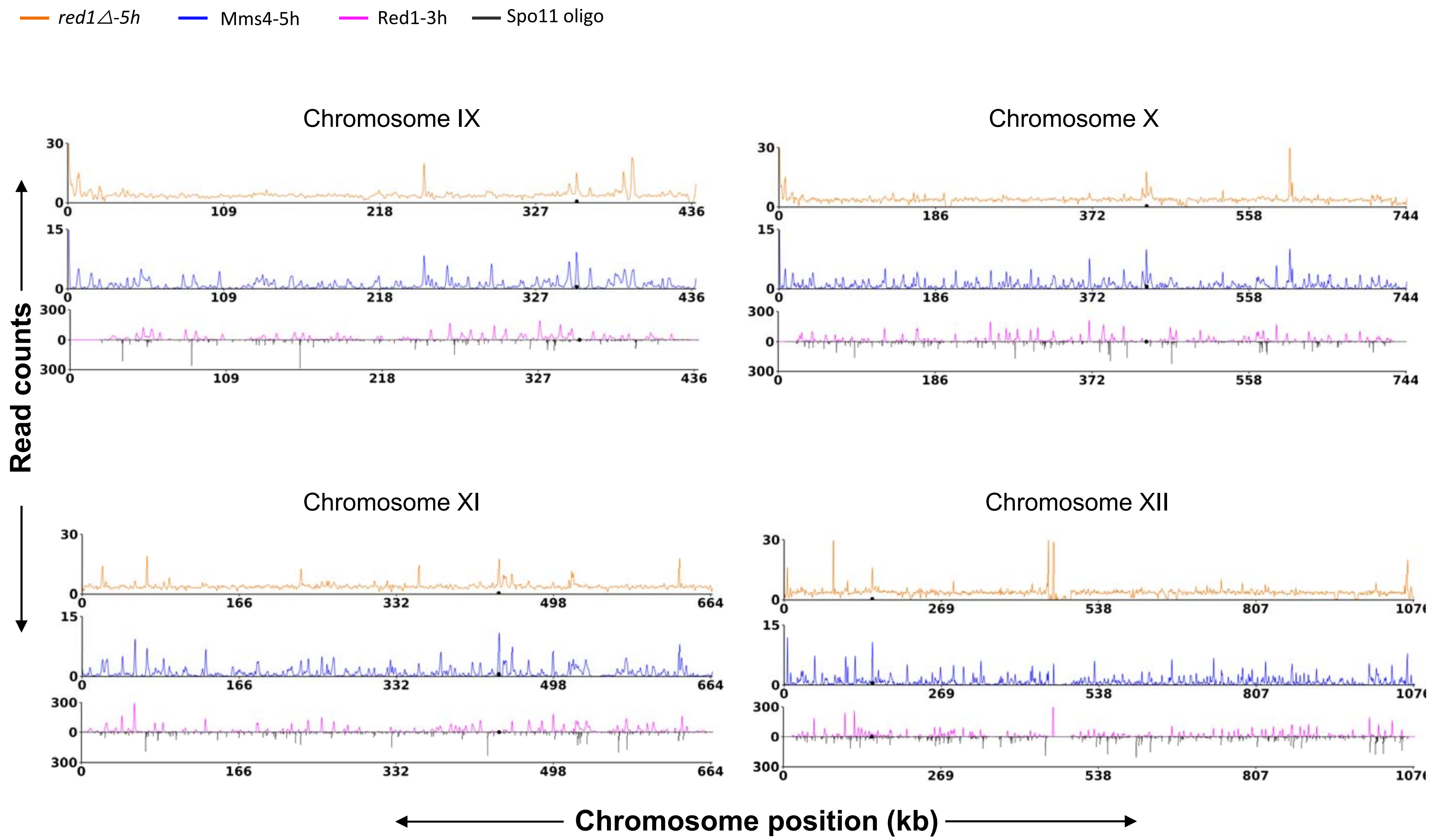

S2 Fig. C) Calibrated ChIP-Seq profile showing Mms4-9xMyc binding for all sixteen chromosomes at 5 hours post meiotic induction in a *red1△* background. Red1 and Spo11 data are from Sun *et al.*, 2015 and Pan *et al.*, 2011 respectively.
The black circle marks the centromere.

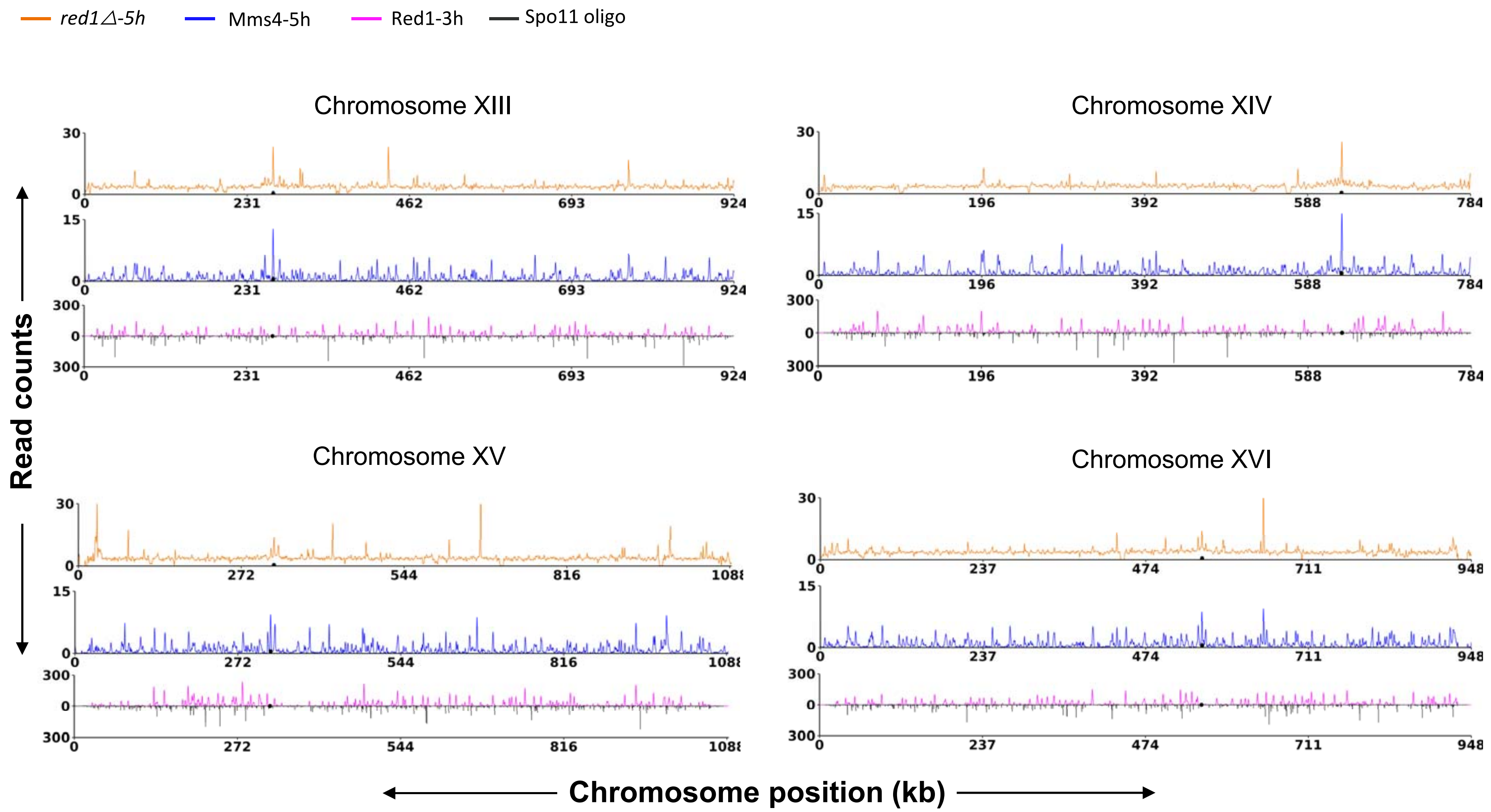

S2 Fig. C) Calibrated ChIP-Seq profile showing Mms4-9xMyc binding for all sixteen chromosomes at 5 hours post meiotic induction in a *red1*△ background. Red1 and Spo11 data are from Sun *et al.*, 2015 and Pan *et al.*, 2011 respectively.
The black circle marks the centromere.

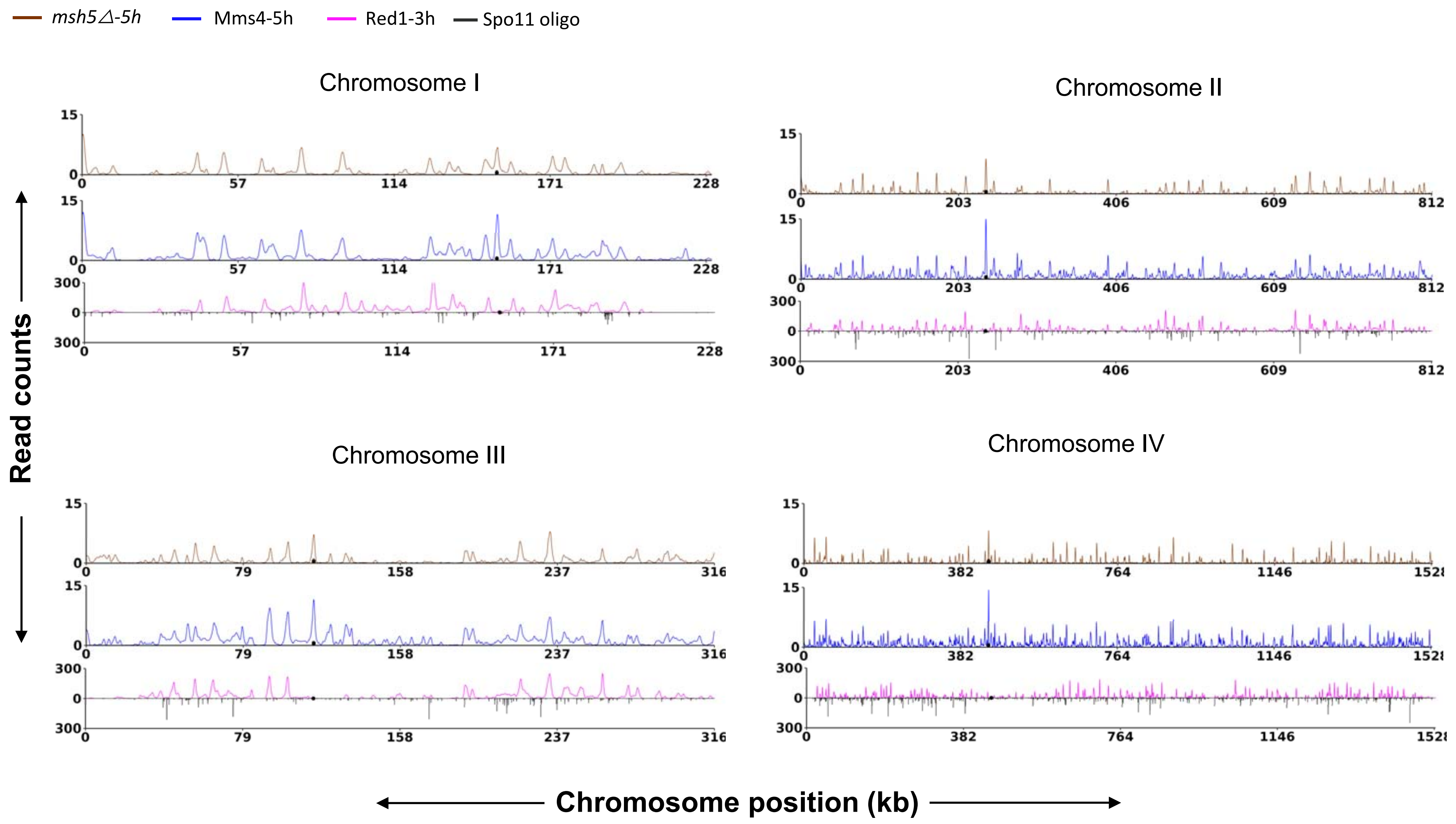

S2 Fig. D) Calibrated ChIP-Seq profile showing Mms4-9xMyc binding for all sixteen chromosomes at 5 hours post meiotic induction in a *msh5*△ background. Red1 and Spo11 data are from Sun *et al.*, 2015 and Pan *et al.*, 2011 respectively.
The black circle marks the centromere.

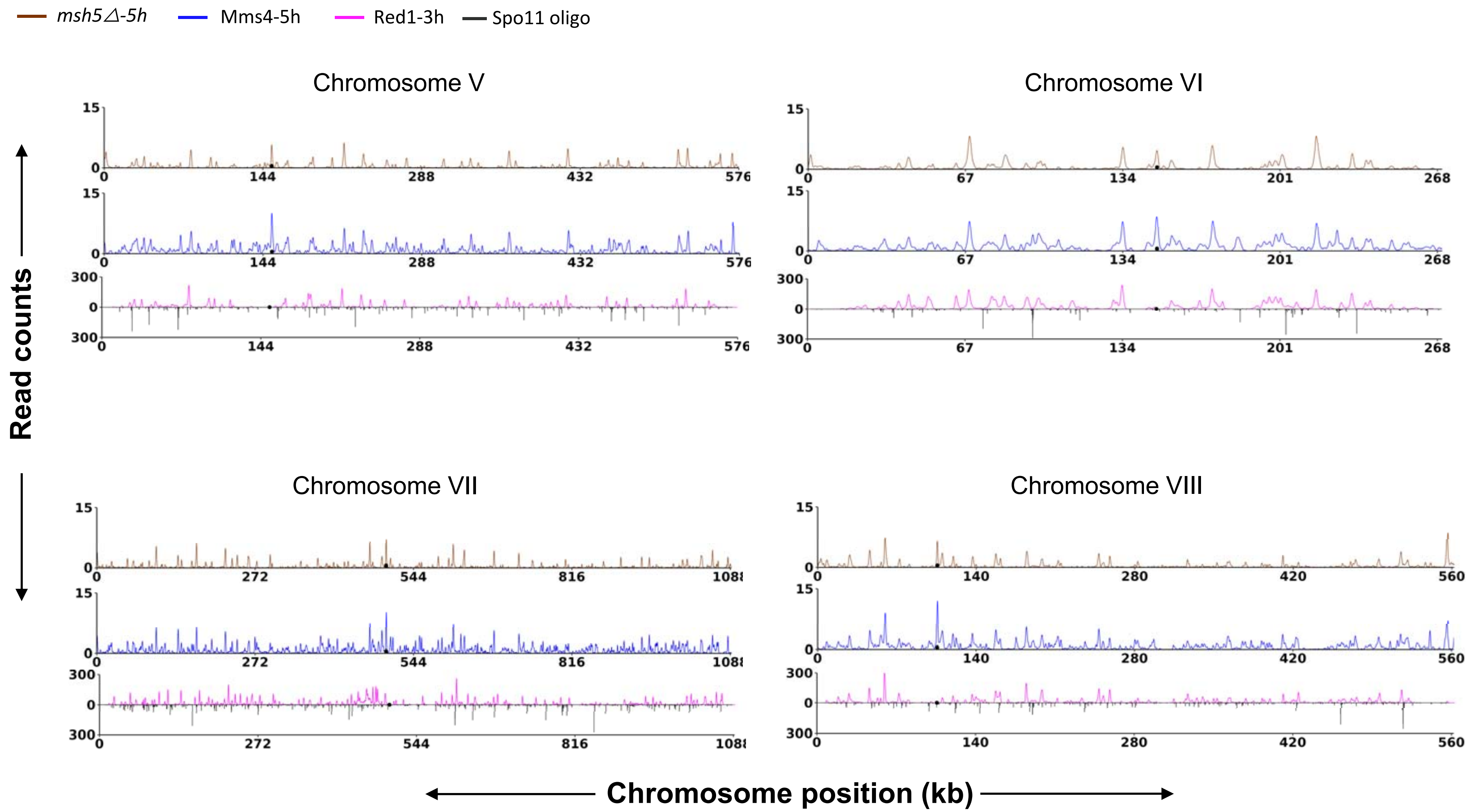

S2 Fig. D) Calibrated ChIP-Seq profile showing Mms4-9xMyc binding for all sixteen chromosomes at 5 hours post meiotic induction in a *msh5*△ background. Red1 and Spo11 data are from Sun *et al.*, 2015 and Pan *et al.*, 2011 respectively.
The black circle marks the centromere.

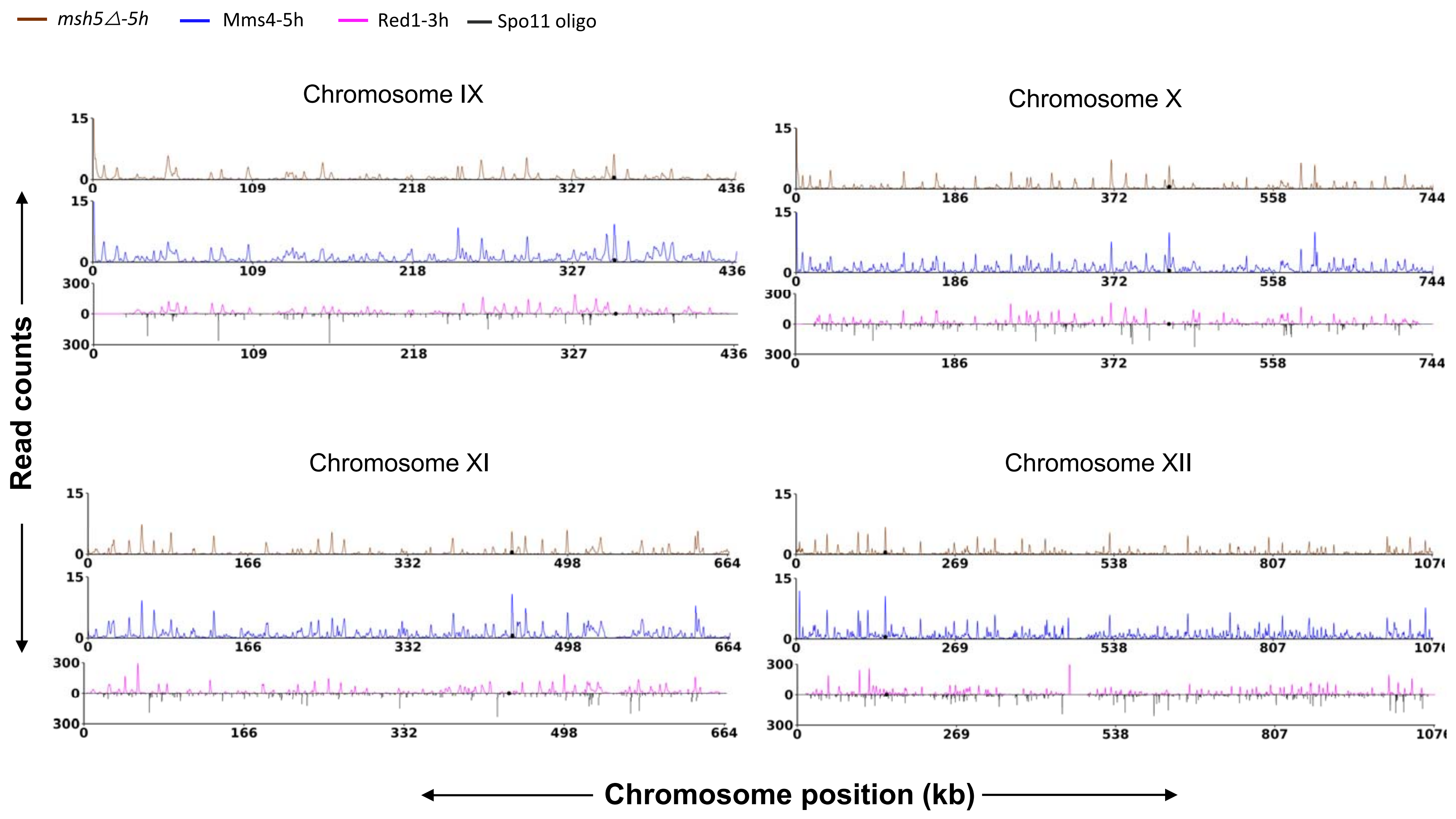

S2 Fig. D) Calibrated ChIP-Seq profile showing Mms4-9xMyc binding for all sixteen chromosomes at 5 hours post meiotic induction in a *msh5Δ* background. Red1 and Spo11 data are from Sun *et al.*, 2015 and Pan *et al.*, 2011 respectively.
The black circle marks the centromere.

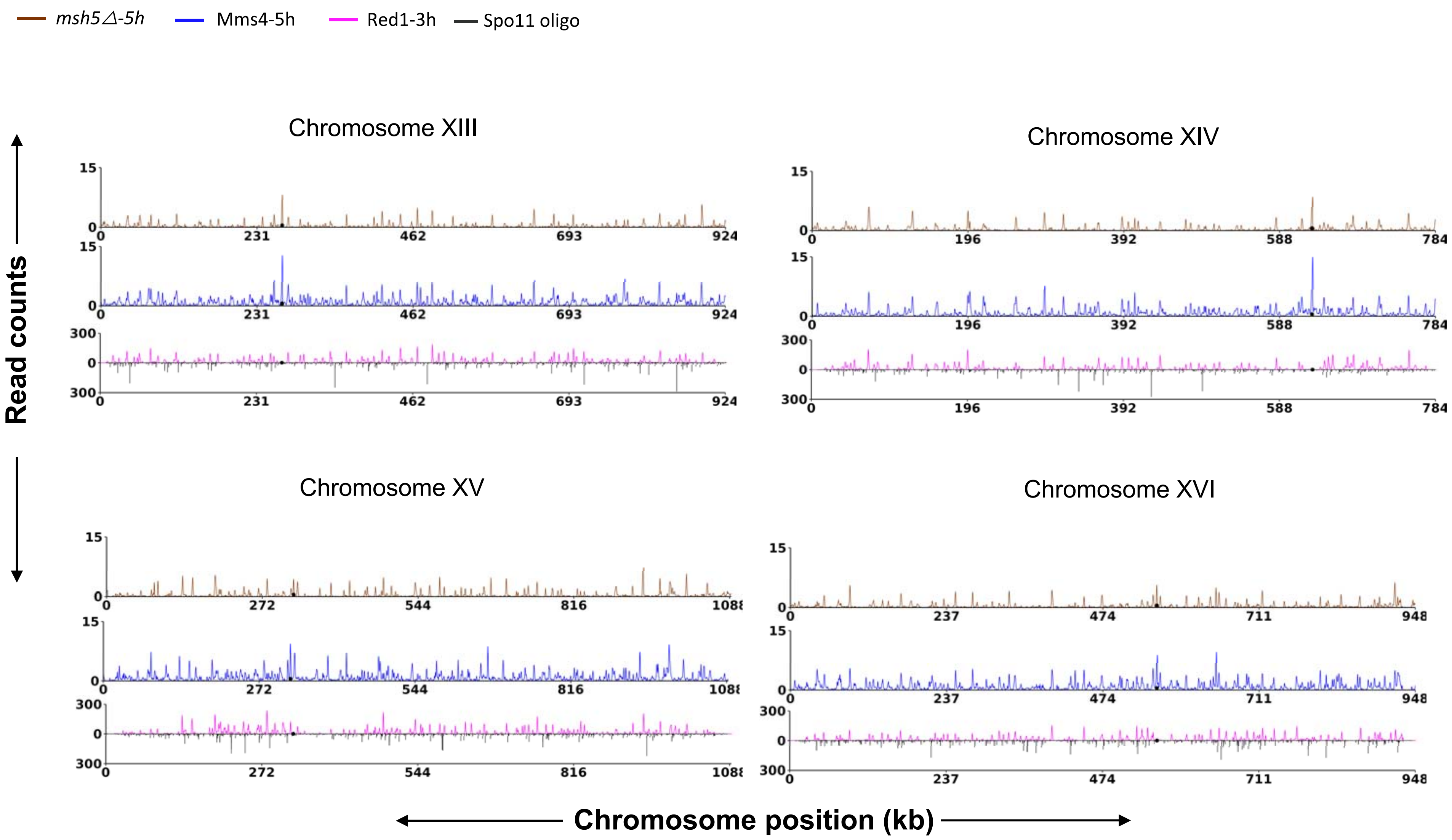

S2 Fig. D) Calibrated ChIP-Seq profile showing Mms4-9xMyc binding for all sixteen chromosomes at 5 hours post meiotic induction in a *msh5△* background. Red1 and Spo11 data are from Sun *et al.*, 2015 and Pan *et al.*, 2011 respectively.
The black circle marks the centromere.

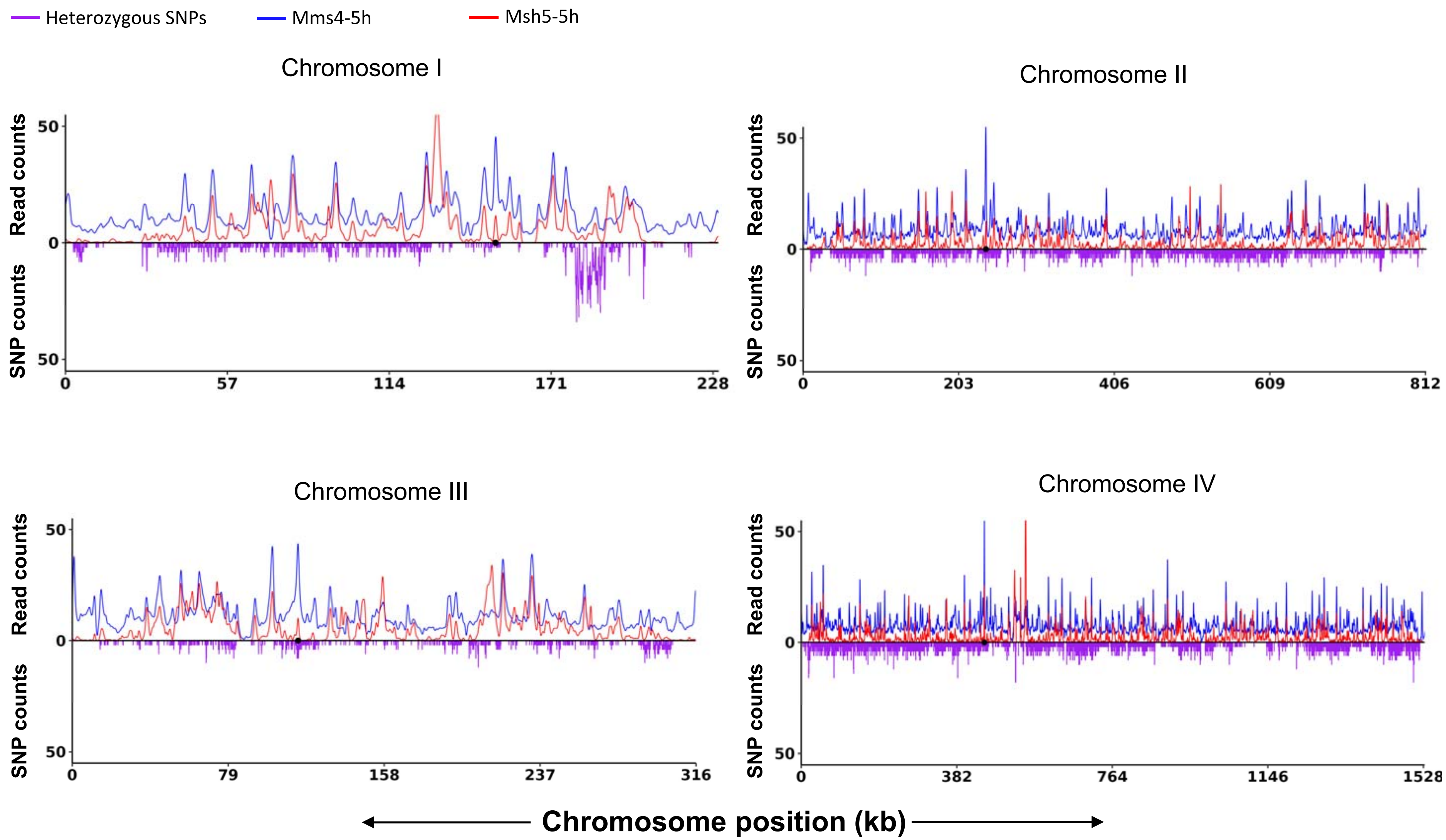

S2 Fig. E) Binding profiles of Mms4 and Msh5 in the S288c-sp/YJM789 hybrid strain for all sixteen chromosomes alongside the corresponding heterozygous SNP density plot. This visualization illustrates the relationship between Mms4 binding and SNP distribution compared to Msh5. For visualization of the heterozygous SNP counts, the S288c genome was partitioned into 100 bp bins and the number of SNPs were counted in those bins and multiplied by 2. Wild type Msh5 binding data were taken from Dash *et al.,* 2024 and Msh5 read counts were divided by 4 for visualization.

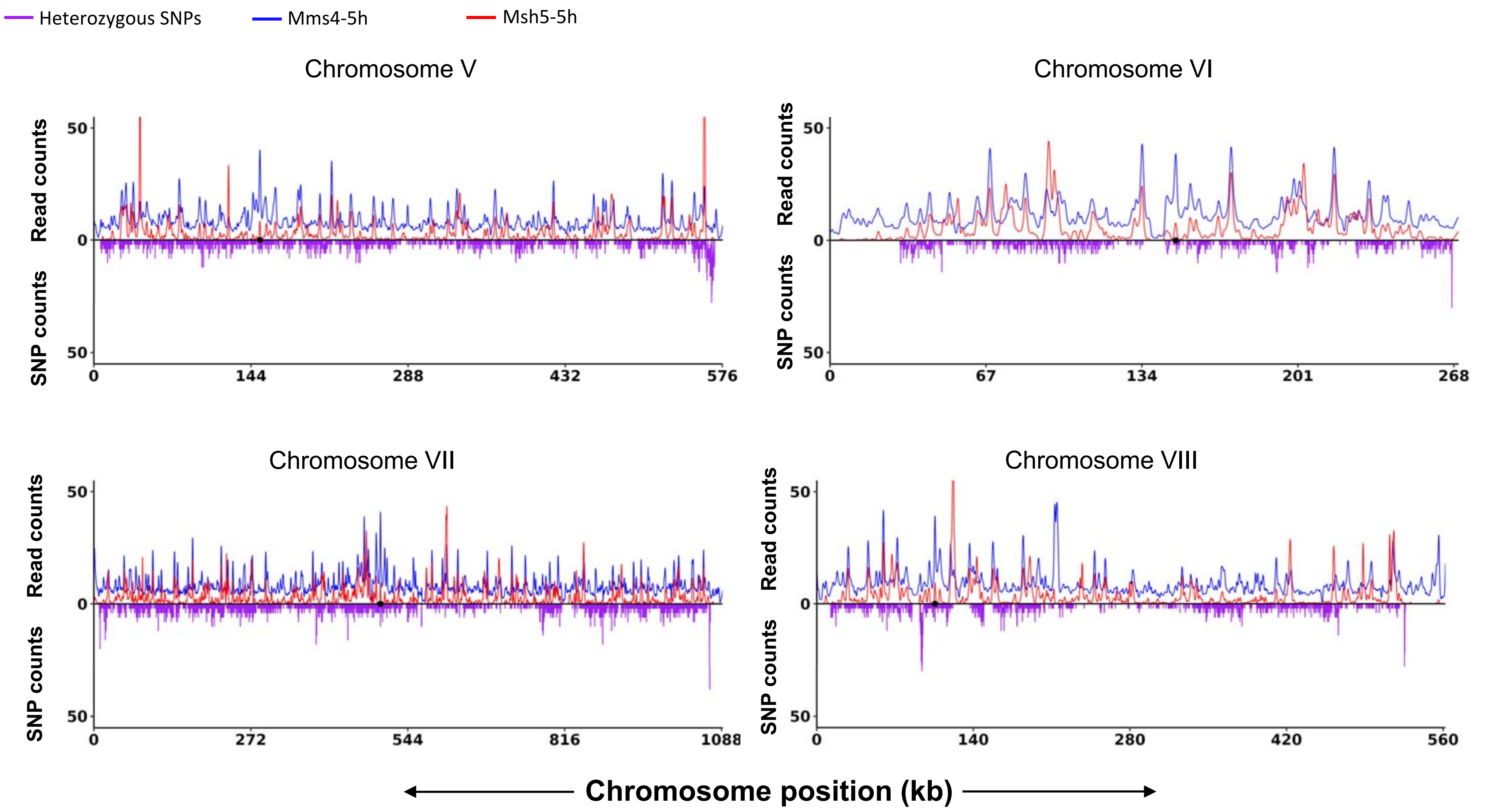

S2 Fig. E) Binding profiles of Mms4 and Msh5 in the S288c-sp/YJM789 hybrid strain for all sixteen chromosomes alongside the corresponding heterozygous SNP density plot. This visualization illustrates the relationship between Mms4 binding and SNP distribution compared to Msh5. For visualization of the heterozygous SNP counts, the S288c genome was partitioned into 100 bp bins and the number of SNPs were counted in those bins and multiplied by 2. Wild type Msh5 binding data were taken from Dash *et al.,* 2024 and Msh5 read counts were divided by 4 for visualization.

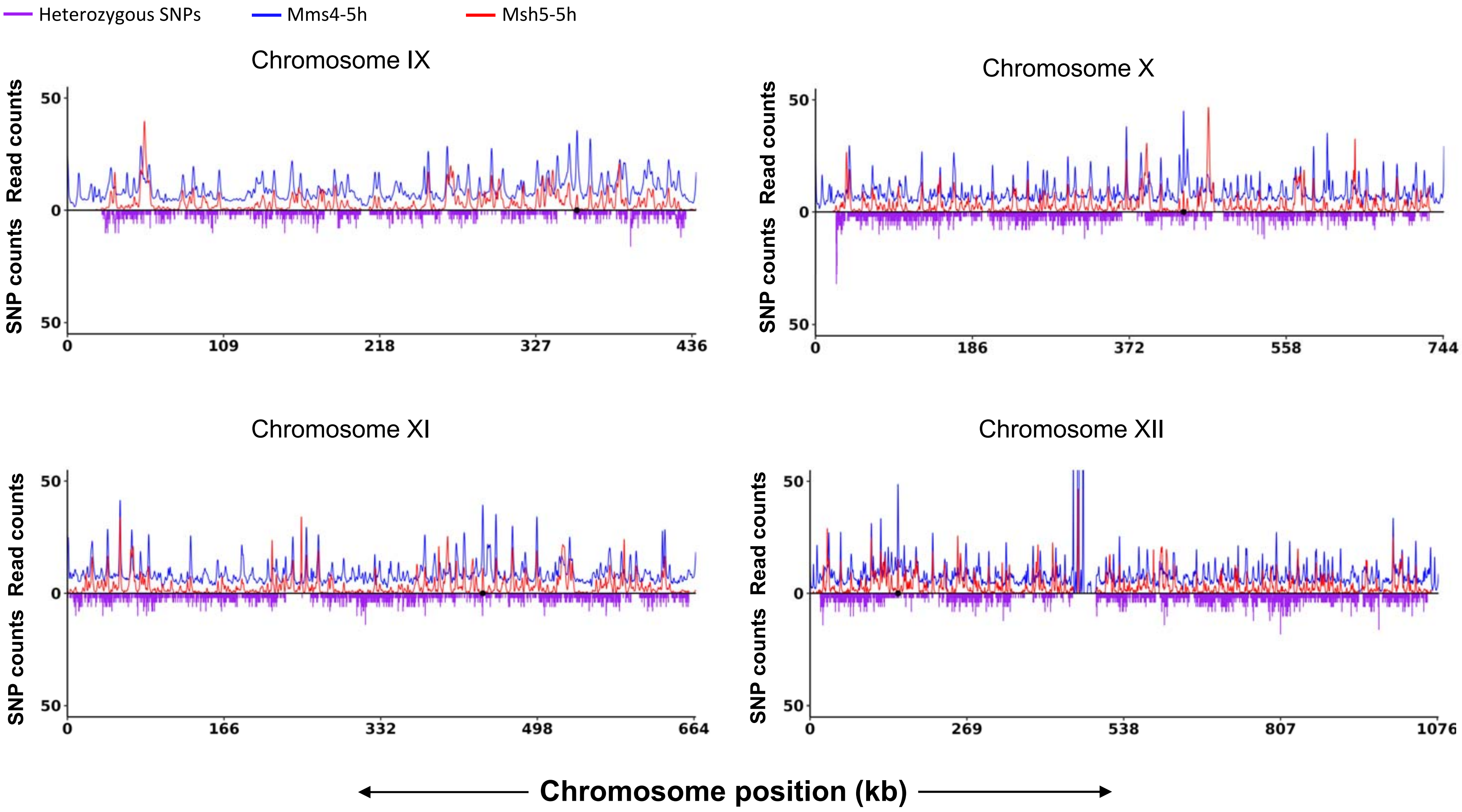

S2 Fig. E) Binding profiles of Mms4 and Msh5 in the S288c-sp/YJM789 hybrid strain for all sixteen chromosomes alongside the corresponding heterozygous SNP density plot. This visualization illustrates the relationship between Mms4 binding and SNP distribution compared to Msh5. For visualization of the heterozygous SNP counts, the S288c genome was partitioned into 100 bp bins and the number of SNPs were counted in those bins and multiplied by 2. Wild type Msh5 binding data were taken from Dash *et al.,* 2024 and Msh5 read counts were divided by 4 for visualization.

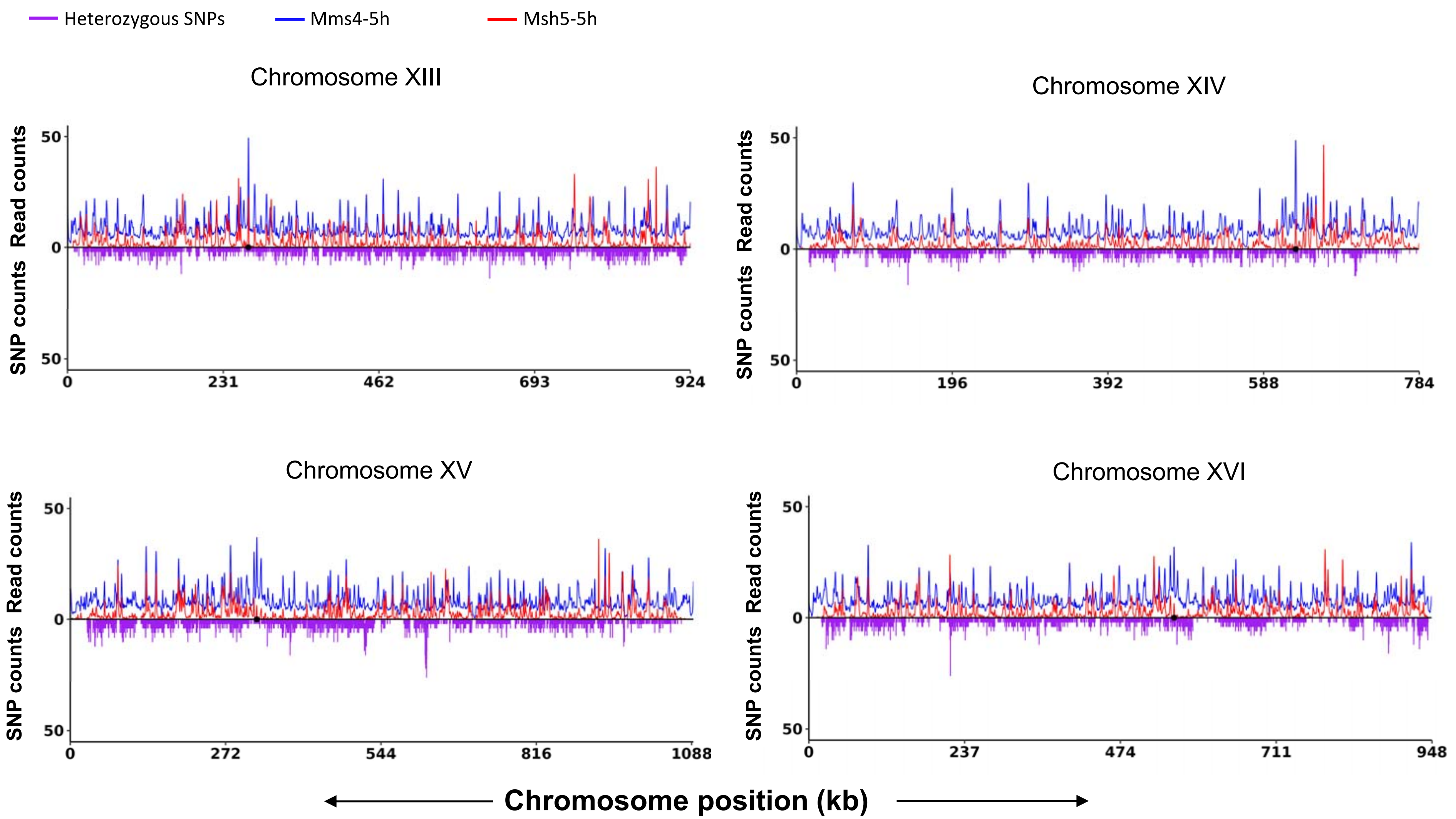

S2 Fig. E) Binding profiles of Mms4 and Msh5 in the S288c-sp/YJM789 hybrid strain for all sixteen chromosomes alongside the corresponding heterozygous SNP density plot. This visualization illustrates the relationship between Mms4 binding and SNP distribution compared to Msh5. For visualization of the heterozygous SNP counts, the S288c genome was partitioned into 100 bp bins and the number of SNPs were counted in those bins and multiplied by 2. Wild type Msh5 binding data were taken from Dash *et al.,* 2024 and Msh5 read counts were divided by 4 for visualization.

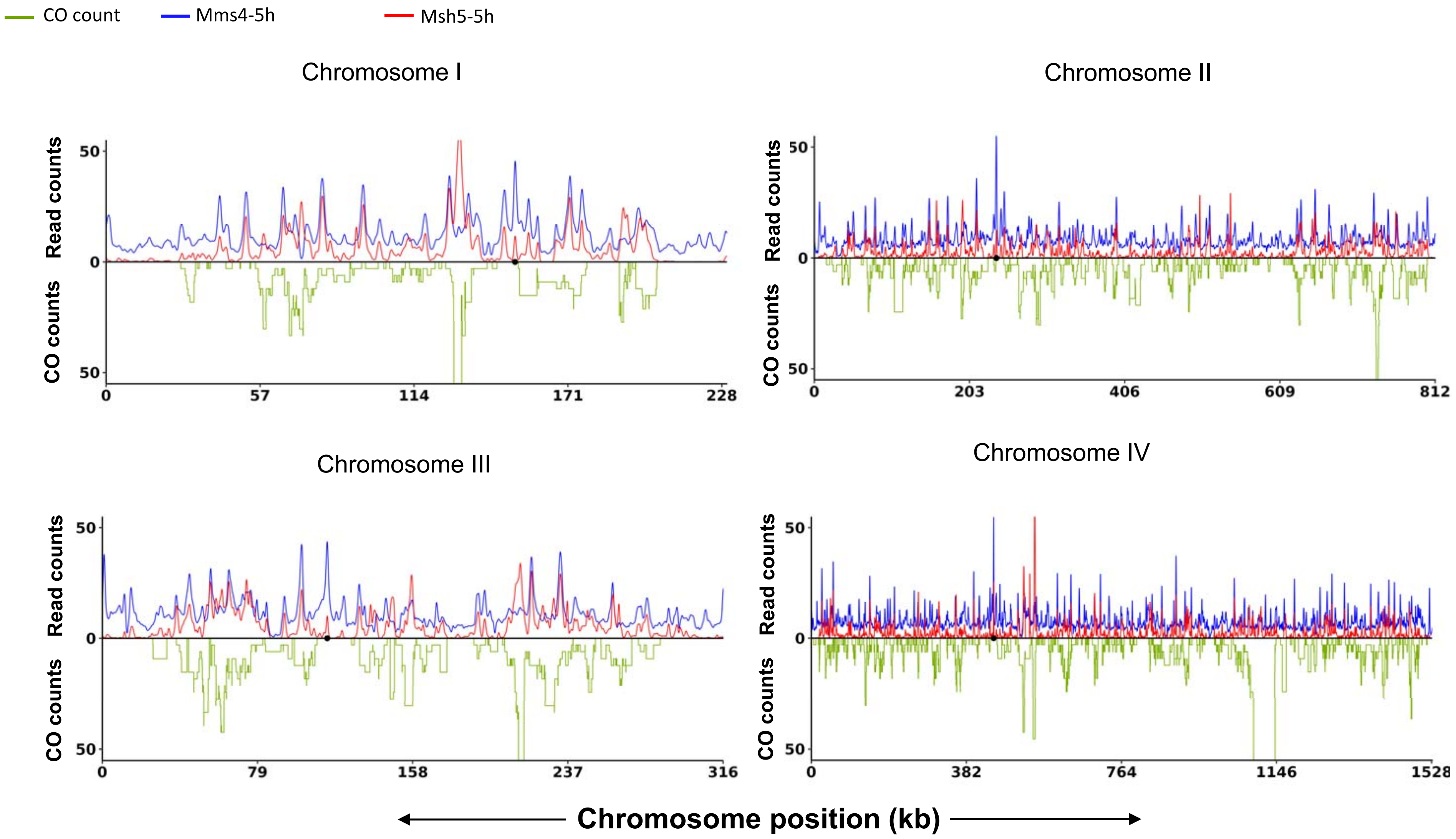

S2 Fig. F) Binding profiles of Mms4 and Msh5 in the S288c-sp/YJM789 hybrid strain for all sixteen chromosomes alongside the corresponding crossover frequency plot. This visualization illustrates the relationship between Mms4 binding and crossover distribution compared to Msh5. Crossover data was obtained from 66 tetrads of the S288c/YJM789 hybrid (Chakraborty *et al.,* 2017). Crossover counts per base were calculated for the S288c genome, and these counts were divided by 66 to get the crossover counts per tetrad for the entire S288c genome. These values were multiplied by 200 for visualization. Wild-type Msh5 binding data were taken from Dash *et al.,* 2024 and Msh5 read counts were divided by 4 for visualization.

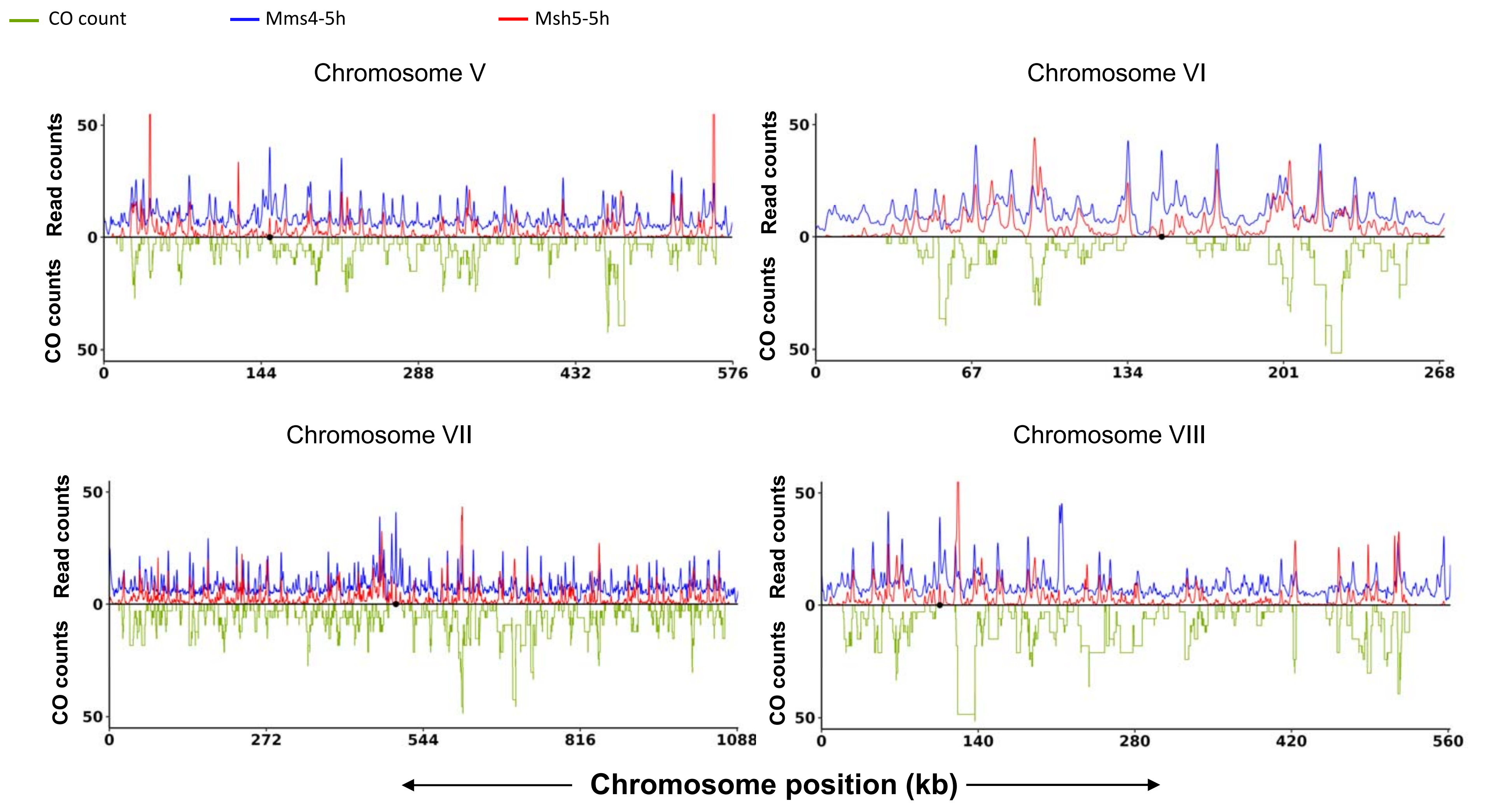

S2 Fig. F) Binding profiles of Mms4 and Msh5 in the S288c-sp/YJM789 hybrid strain for all sixteen chromosomes alongside the corresponding crossover frequency plot. This visualization illustrates the relationship between Mms4 binding and crossover distribution compared to Msh5. Crossover data was obtained from 66 tetrads of the S288c/YJM789 hybrid (Chakraborty *et al.,* 2017). Crossover counts per base were calculated for the S288c genome, and these counts were divided by 66 to get the crossover counts per tetrad for the entire S288c genome. These values were multiplied by 200 for visualization. Wild-type Msh5 binding data were taken from Dash *et al.,* 2024 and Msh5 read counts were divided by 4 for visualization.

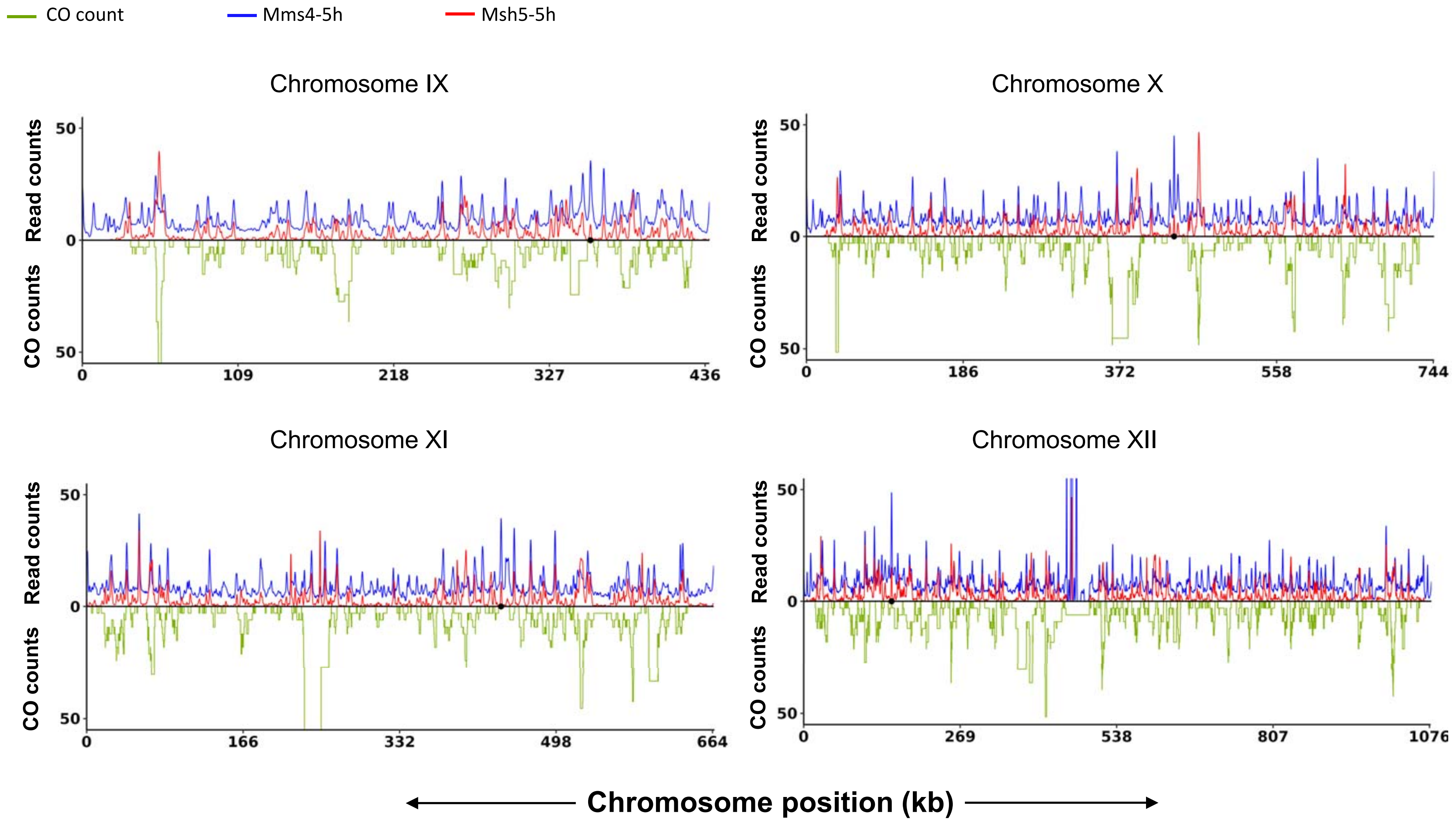

S2 Fig. F) Binding profiles of Mms4 and Msh5 in the S288c-sp/YJM789 hybrid strain for all sixteen chromosomes alongside the corresponding crossover frequency plot. This visualization illustrates the relationship between Mms4 binding and crossover distribution compared to Msh5. Crossover data was obtained from 66 tetrads of the S288c/YJM789 hybrid (Chakraborty *et al.,* 2017). Crossover counts per base were calculated for the S288c genome, and these counts were divided by 66 to get the crossover counts per tetrad for the entire S288c genome. These values were multiplied by 200 for visualization. Wild-type Msh5 binding data were taken from Dash *et al.,* 2024 and Msh5 read counts were divided by 4 for visualization.

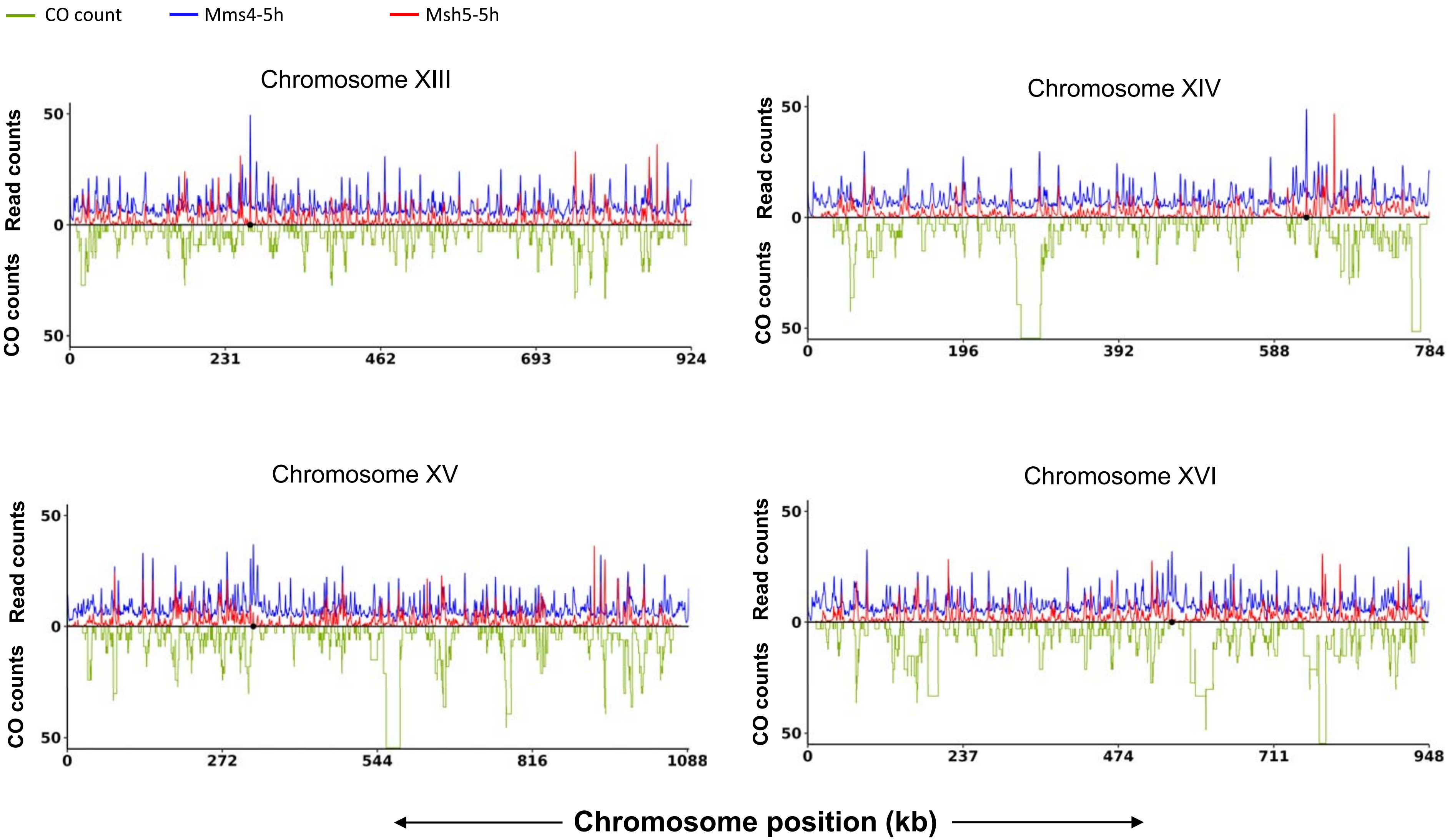

S2 Fig. F) Binding profiles of Mms4 and Msh5 in the S288c-sp/YJM789 hybrid strain for all sixteen chromosomes alongside the corresponding crossover frequency plot. This visualization illustrates the relationship between Mms4 binding and crossover distribution compared to Msh5. Crossover data was obtained from 66 tetrads of the S288c/YJM789 hybrid (Chakraborty *et al.*, 2017). Crossover counts per base were calculated for the S288c genome, and these counts were divided by 66 to get the crossover counts per tetrad for the entire S288c genome. These values were multiplied by 200 for visualization. Wild-type Msh5 binding data were taken from Dash *et al.,* 2024 and Msh5 read counts were divided by 4 for visualization.
