# Supplementary material for: Mms4 chromosomal association reveals functional relationships between meiotic crossover pathways in budding yeast
Source: PLoS Genet. 2026 Mar 30;22(3):e1012097. doi: 10.1371/journal.pgen.1012097 (PMC13046247; doi:10.1371/journal.pgen.1012097)
Supplement: S3 Fig — Black circles show the centromere on each chromosome. B) Bar plot representing distance from the centromere for DSB hotspots bound uniquely by Mms4 or Msh5. ns indicates non-significant p value (Wilcoxon-Rank Sum test). (PDF) [file pgen.1012097.s003.pdf]

A

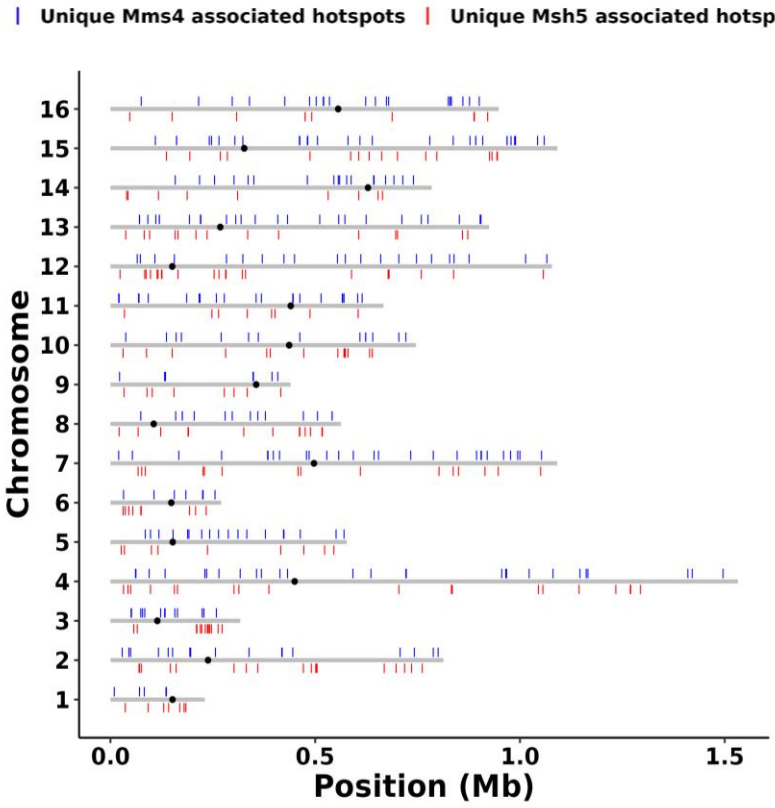

B

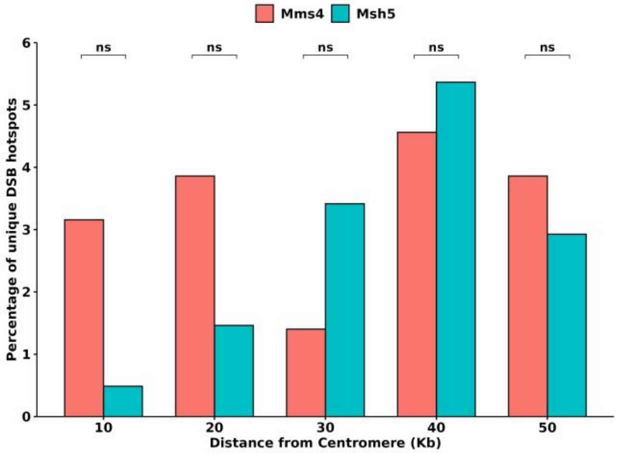

S3 Fig. A) Locations of DSB hotspots uniquely associated with Mms4 or Msh5. Black circles show the centromere on each chromosome. **B) Bar plot representing distance from the centromere for DSB hotspots bound uniquely by Mms4 or Msh5.** ns indicates non-significant p value (Wilcoxon-Rank Sum test).
