# Supplementary material for: Mms4 chromosomal association reveals functional relationships between meiotic crossover pathways in budding yeast
Source: PLoS Genet. 2026 Mar 30;22(3):e1012097. doi: 10.1371/journal.pgen.1012097 (PMC13046247; doi:10.1371/journal.pgen.1012097)
Supplement: S4 Fig — Western blot analysis of Mms4 expression from 0 to 8h post onset of meiosis in A) spo11△, B) red1△ and C) msh5△. For comparison, Mms4 expression in the wild-type strain (0-9h) is also shown (D). Pgk1 expression is shown as a control in all of the above blots. (PDF) [file pgen.1012097.s004.pdf]

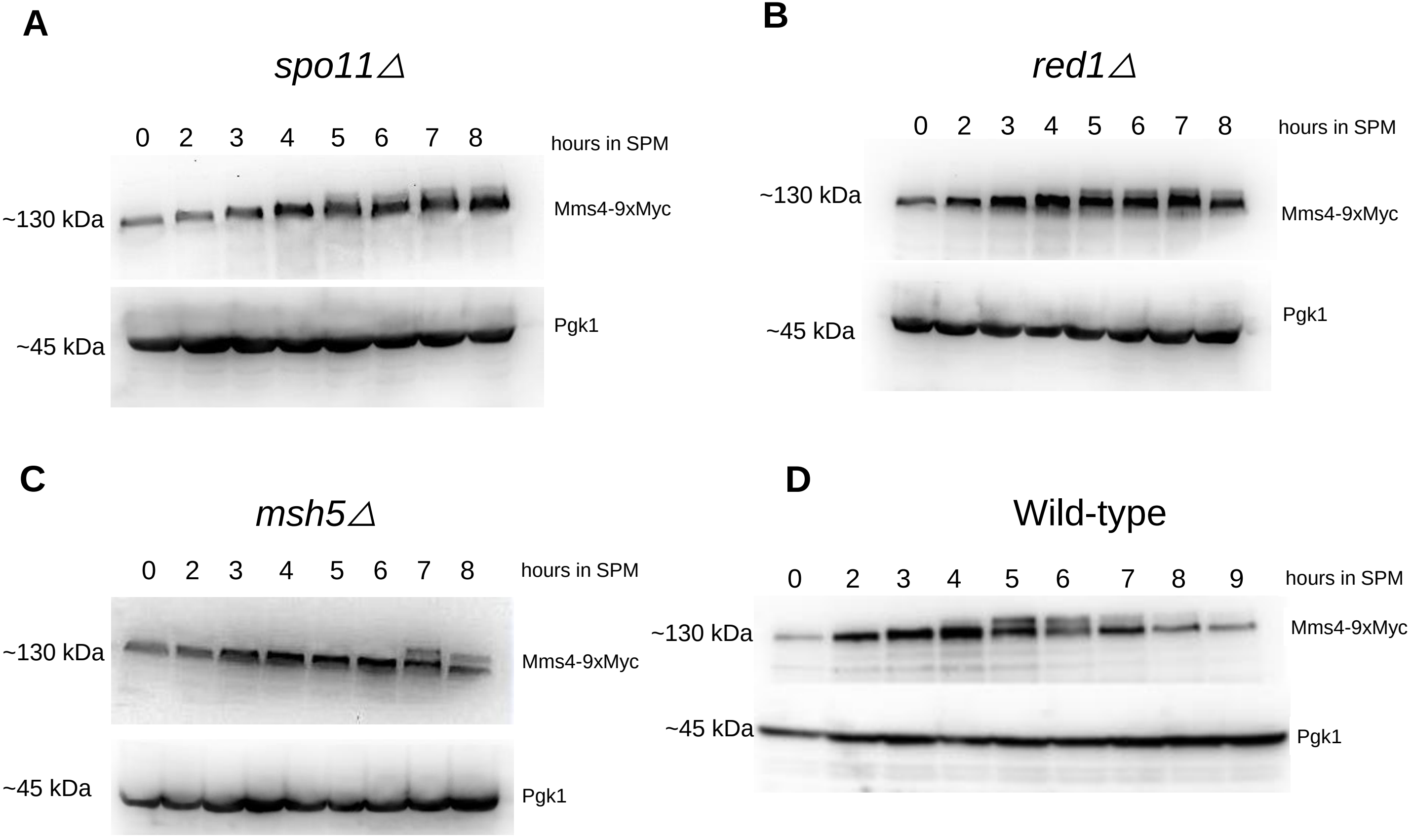

S4 Fig. Mms4 expression analysis in mutants. Western blot analysis of Mms4 expression from 0 to 8h post onset of meiosis in **A)** *spo11△,* **B)** *red1△* and **C)** *msh5△*. For comparison, Mms4 expression in the wild-type strain (0-9h) is also shown **(D)**. Pgk1 expression is shown as a control in all of the above blots.
