# Supplementary material for: Mms4 chromosomal association reveals functional relationships between meiotic crossover pathways in budding yeast
Source: PLoS Genet. 2026 Mar 30;22(3):e1012097. doi: 10.1371/journal.pgen.1012097 (PMC13046247; doi:10.1371/journal.pgen.1012097)
Supplement: S5 Fig — Immunoblot analysis of Mms4-9xMyc ChIP using anti-Myc antibody in synchronised meiotic cultures of A) red1△, spo11△ and B) msh5△ mutants. A) Lanes 1, 2 and 3 for red1△ and lanes 4, 5, and 6 for spo11△ strains indicate lysate before incubation with magnetic beads, lysate after incubation with beads, and eluate fraction. M represents marker. B) Lanes 1, 2 and 3 for msh5△ strains indicate lysate before incubation with magnetic beads, lysate after incubation with beads, and eluate fraction. (PDF) [file pgen.1012097.s005.pdf]

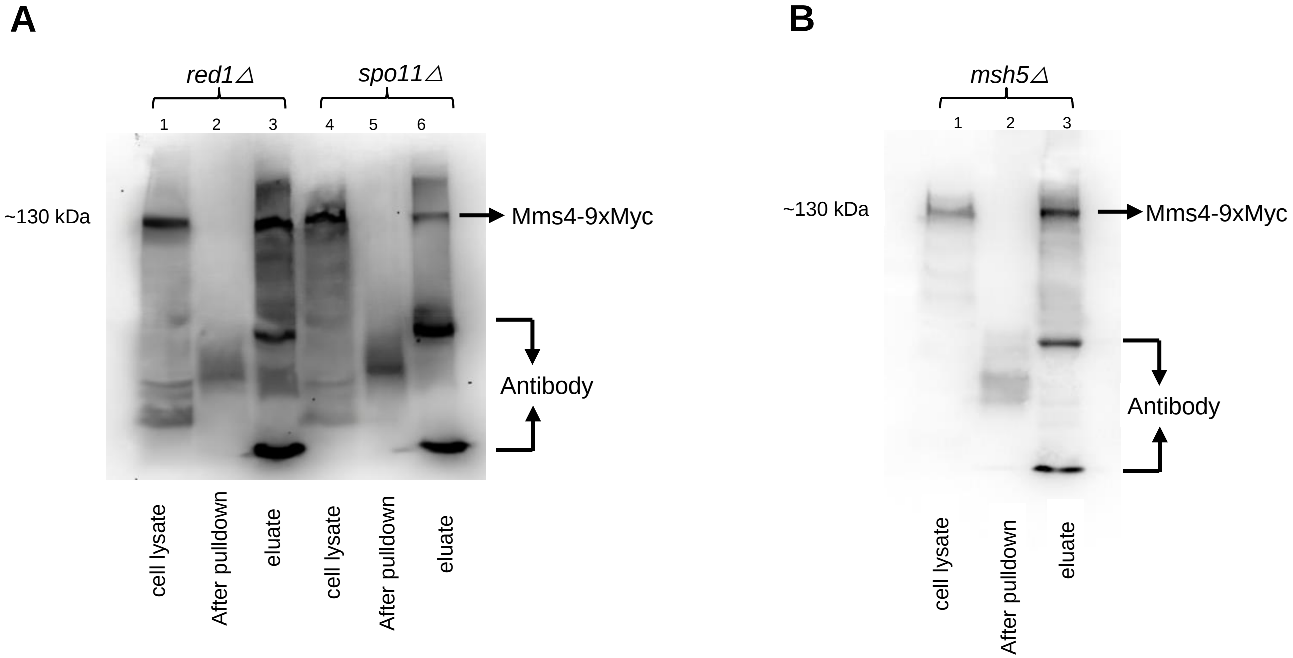

S5 Fig. Mms4 ChIP in mutants. Immunoblot analysis of Mms4-9xMyc ChIP using anti-Myc antibody in synchronised meiotic cultures of **A)** *red1△, spo11△* and **B)** *msh5△* mutants. **A)** Lanes 1, 2 and 3 for *red1△* and lanes 4, 5, and 6 for *spo11△* strains indicate lysate before incubation with magnetic beads, lysate after incubation with beads, and eluate fraction. M represents marker. **B)** Lanes 1, 2 and 3 for *msh5△* strains indicate lysate before incubation with magnetic beads, lysate after incubation with beads, and eluate fraction.
