# Supplementary material for: Mms4 chromosomal association reveals functional relationships between meiotic crossover pathways in budding yeast
Source: PLoS Genet. 2026 Mar 30;22(3):e1012097. doi: 10.1371/journal.pgen.1012097 (PMC13046247; doi:10.1371/journal.pgen.1012097)
Supplement: S6 Fig — Data are from two independent biological replicates. (PDF) [file pgen.1012097.s006.pdf]

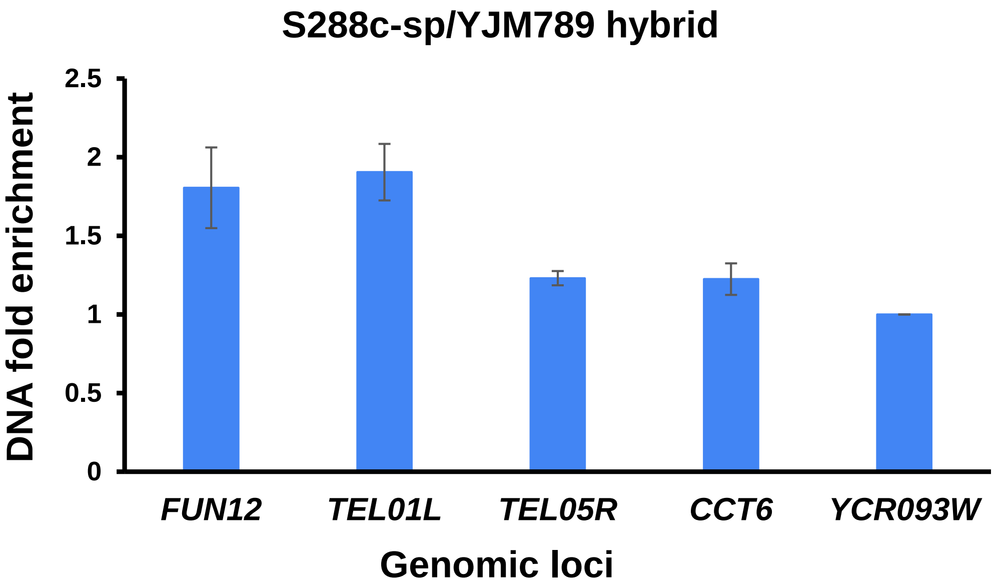

S6 Fig. ChIP-qPCR analysis showing Mms4 enrichment at representative DSB hotspots (*FUN12*, *TEL01L*, *TEL05R*, *CCT6*) with reference to the DSB coldspot (*YCR093W*) in the S288c-sp/YJM789 hybrid at 5h time point. Data are from two independent biological replicates.
