# Supplementary material for: Mms4 chromosomal association reveals functional relationships between meiotic crossover pathways in budding yeast
Source: PLoS Genet. 2026 Mar 30;22(3):e1012097. doi: 10.1371/journal.pgen.1012097 (PMC13046247; doi:10.1371/journal.pgen.1012097)
Supplement: S1 Table — (PDF) [file pgen.1012097.s007.pdf]

S1 Table. Strains used in this study.

Number	Strain	Genotype	Source
NHY1162	SK1	*MATα ho::hisG,leu::hisG,ura3(Δsma-pst),his4-X::LEU2-(NgoM IV---URA3)*	Martini *et al.*, 2006
NHY1168	SK1	*MATa ho::hisG,leu::hisG,ura3(Δsma-pst),HIS4::LEU2-(BamH1)*	Martini *et al.*, 2006
KTY1188	SK1	*MATα ho::hisG,leu::hisG,ura3(Δsma-pst),his4-X::LEU2-(NgoM IV---URA3) , MMS4-9xMyc::hphNT1*	This study
KTY1189	SK1	*MATa ho::hisG,leu::hisG,ura3(Δsma-pst),HIS4::LEU2-(BamH1) , MMS4-9xMyc::hphNT1*	This study
KTY1190	SK1	*MATα ho::hisG, leu::hisG, ura3(Δsma-pst), his4-X::LEU2-(NgoM IV)---URA3, spo11Δ::kanMX4, MMS4-9xMyc::hphNT1*	This study
KTY1191	SK1	*MATa ho::hisG, leu::hisG, ura3(Δsma-pst), HIS4::LEU2-(BamH1), spo11Δ::kanMX4, MMS4-9xMyc::hphNT1*	This study
KTY1192	SK1	*MATα ho::hisG, leu::hisG, ura3(Δsma-pst), his4-X::LEU2-(NgoM IV)---URA3, red1Δ::kanMX4, MMS4-9xMyc::hphNT1*	This study
KTY1193	SK1	*MATa ho::hisG, leu::hisG, ura3(Δsma-pst), HIS4::LEU2-(BamH1), red1Δ::kanMX4, MMS4-9xMyc::hphNT1*	This study
KTY1194	SK1	*MATα ho::hisG, leu::hisG, ura3(Δsma-pst), his4-X::LEU2-(NgoM IV)---URA3, msh5Δ::natMX4, MMS4-9xMyc::hphNT1*	This study
KTY1195	SK1	*MATa ho::hisG, leu::hisG, ura3(Δsma-pst), HIS4::LEU2-(BamH1), msh5Δ::natMX4, MMS4-9xMyc::hphNT1*	This study
KTY327	S288c-sp	*MATa RME1(ins- 308A) TAO3(E1493Q) MKT1(D30G)*	Dash *et al.*, 2024
KTY83	YJM789	*MATα ho::hisG lys2 cyh*	Mancera *et al.*, 2008
KTY1196	S288c-sp	*MATa RME1(ins- 308A) TAO3(E1493Q) MKT1(D30G) MMS4-9xMyc::hphNT1*	This study
KTY1197	YJM789	*MATα ho::hisG lys2 cyh, MMS4-9xMyc::hphNT1*	This study
KTY1198	IFO1815	*S. mikatae MMS4*	Kellis *et al.*, 2003

KTY1199	IFO1815	*S. mikatae MMS4-9xMyc::hphNT1*	This study

Mancera, E., Bourgon, R., Brozzi, A., Huber, W., & Steinmetz, L.M. (2008). High-resolution mapping of meiotic crossovers and non-crossovers in yeast. *Nature*, 454**:** 479-485.

Kellis, M., Patterson, N., Endrizzi, M., Birren, B., & Lander, E.S. (2003). Sequencing and comparison of yeast species to identify genes and regulatory elements. *Nature*, 423: 241–254.

Martini, E., Diaz, R.L., Hunter, N., & Keeney, S. (2006). Crossover homeostasis in yeast meiosis. *Cell*, 126: 285-295.

Dash, S., Joshi, S., Pankajam, A. V., Shinohara, A., & Nishant, K. T. (2024). Heterozygosity alters Msh5 binding to meiotic chromosomes in the baker's yeast. *Genetics*, 226(3), iyad214.
